# Robust and efficient fixed-point algorithm for the inverse elastostatic problem to identify myocardial passive material parameters and the unloaded reference configuration

**DOI:** 10.1016/j.jcp.2022.111266

**Published:** 2022-08

**Authors:** Laura Marx, Justyna A. Niestrawska, Matthias A.F. Gsell, Federica Caforio, Gernot Plank, Christoph M. Augustin

**Affiliations:** a Gottfried Schatz Research Center for Cell Signaling, Metabolism and Aging - Division of Biophysics, Medical University of Graz, Graz, Austria; b Institute of Mathematics and Scientific Computing, University of Graz, Graz, Austria; c BioTechMed-Graz, Graz, Austria

**Keywords:** Unloaded reference configuration, Parameter estimation, Cardiac mechanics, Passive biomechanical properties, Patient-specific modeling

## Abstract

Image-based computational models of the heart represent a powerful tool to shed new light on the mechanisms underlying physiological and pathological conditions in cardiac function and to improve diagnosis and therapy planning. However, in order to enable the clinical translation of such models, it is crucial to develop personalized models that are able to reproduce the physiological reality of a given patient. There have been numerous contributions in experimental and computational biomechanics to characterize the passive behavior of the myocardium. However, most of these studies suffer from severe limitations and are not applicable to high-resolution geometries. In this work, we present a novel methodology to perform an automated identification of *in vivo* properties of passive cardiac biomechanics. The highly-efficient algorithm fits material parameters against the shape of a patient-specific approximation of the end-diastolic pressure-volume relation (EDPVR). Simultaneously, an unloaded reference configuration is generated, where a novel line search strategy to improve convergence and robustness is implemented. Only clinical image data or previously generated meshes at one time point during diastole and one measured data point of the EDPVR are required as an input. The proposed method can be straightforwardly coupled to existing finite element (FE) software packages and is applicable to different constitutive laws and FE formulations. Sensitivity analysis demonstrates that the algorithm is robust with respect to initial input parameters.

## 1 Introduction

Computational models of the heart are widely recognized as a powerful tool for the quantitative analysis of cardiac function. Their ability to explore mechanistic relationships of all variables of interest at high spatio-temporal resolution has turned computational models into an important, if not indispensable, adjunct in any basic research study. More recently though, driven by advances in medical imaging and simulation technologies, a translational trend has emerged that is geared towards turning cardiac modeling from a research tool into a clinical modality for diagnosis, stratification, and therapy planning [46,76,60].

However, unlike in basic research applications where a representative "one heart fits all" model is suitable to investigate generic mechanisms, in clinical scenarios the use of patient-specific models is of fundamental importance. Computational models must be built to represent the physiological reality of a given patient to provide a basis for therapeutic decisions for the specific patient, not for a representative average patient.

This process of minimizing the difference between the predictions of a computational model and clinical observations, referred to as "model personalization" or "digital twinning", is challenging to achieve [59]. Cardiac function emerges as a complex interplay between different physics — electrophysiology, mechanics, and hemodynamics. While accurate mechanistic computational models of each of these physical components exist and anatomically accurate multiphysics models of the heart, e.g., [6,50,80], are considered to be the state-of-the-art, they comprise a larger number of parameters, most of which cannot be observed *in vivo* or not at all. Thus, model parameters have to be inferred indirectly from clinical observable quantities which are, in general, afflicted with a significant degree of observational and residual uncertainties [52,51].

Integral model components in modeling cardiac function are constitutive relations which characterize the passive biome-chanical behavior of myocardial tissue. The development and parameterization of these models is an active area of research, see [10] and references therein. As microstructural material components along local material axes determine the orthotropic behavior of the myocardium, recent experimental research has focused on multiaxial mechanical testing and microscopical investigations of the underlying structure [17,77,81]. These experiments are usually performed under artificial *ex vivo* conditions using excised tissue specimen from specific locations within the myocardium. While these experiments are an indispensable source for informing modeling, their inherent underlying limitations and uncertainties should be kept in mind when incorporating such data into models. The tissue is usually excised during autopsies within 12h of death and then stored in a suitable solution until testing. The testing should ideally take place immediately, but, in practice, may be postponed for weeks, although it has been shown for collageneous tissues that changes in microstructure and mechanics occur in as little as 48h after removal from the body [36]. Available material parameters are usually fitted to a normalized stress-strain curve obtained from multiple patients and used to represent the overall passive mechanical behavior of the ensemble. It is known though that tissue properties vary to a significant extent between individuals and throughout the myocardium. Additionally, even if orthotropic, microstructurally-based models are utilized, the underlying orthotropic fiber and sheet arrangement in the myocardium cannot be determined with certainty for individuals. To achieve translation it is therefore key to develop a methodology that facilitates the efficient, robust and, ideally, automated identification of model parameters from clinical observations *in vivo* [6].

A method towards *in vivo* passive parameter estimation was proposed by Augenstein et al. [5]. They utilized an *ex vivo* experimental method based on the combination of tagged magnetic resonance imaging (MRI) and simultaneous pressure recordings to estimate passive material properties in arrested pig hearts. The fiber architecture was incorporated from diffusion tensor MRI and the findings were validated against a silicon gel phantom with known material properties, proving that cardiac MRI can be used to extract meaningful material properties. Further work on isolated hearts combined with FE analysis was then conducted by e.g. Nair et al. [56], where an *ex vivo* rabbit left ventricular (LV) model was used for strain matching.

To take a step towards the translation of passive material identification into the clinics, *in vivo* MRI combined with *ex vivo* diffusion tensor MRI was carried out in heart failure patients [85] and, using a sequential quadratic programming optimization technique [12], passive material parameters were identified [86]. These studies used either transversely isotropic laws such as the Guccione law [32] or orthotropic laws such as the Holzapfel-Ogden (HO) model [39]. Further, initial parameter sets fitted to *ex vivo* experimental data (e.g. [33]) are taken and either only the isotropic parameter is varied [86,4,21] or two scaling parameters [58,46,72,64] are introduced to preserve the overall orthotropy of the material parameters. Additionally, material parameters of these laws are often correlated [70], and hence the unique identification poses a non-trivial problem.

Early studies attempted to identify material parameters based on non-invasive imaging of strain via MRI [57,92]. However, *in vivo* MRI strain measurements have major caveats as they vary among methods, modalities, and software version and mostly lack proper validation [2,69]. In addition, MRI strains are not usually acquired during routine clinical exams, and hence pressure-volume (PV) relations are more commonly used to estimate material parameters. There are two common fitting targets used in literature: fitting to displacement curves obtained from several frames during diastole or approximation of the empirical EDPVR by a power law as proposed by Klotz et al. [45].

Inverse estimation of patient-specific parameters is now widely used, where parameters of a forward problem are tuned to match PV-curves and motion fields available from clinical imaging [24], a method which was first used by Ghista et al. [28]. Xi et al. [89,90] utilized a reduced-order unscented Kalman filter [54] and later 3D tagged MRI to simulate unloading and estimate parameters in a reformulated Guccione law to overcome the problem of non-unique constitutive parameters. They used an early diastolic frame as the reference configuration and 29 frames per cycle for the fitting. Asner et al. [4] jointly estimated the reference state and passive parameters using deflation, considering a parameter sweep consisting of 25 simulations to assess the parameter space and find the minimum of the objective function. Gao et al. [24] used the first frame of early diastole as the unloaded reference configuration and assumed a population-based end-diastolic pressure, ped, of 8 mmHg as no pressure recordings were available. They proposed a multi-step non-linear least-squared optimization procedure for the inverse estimation of all parameters of the HO law. The performance of multiple constitutive laws were compared by Hadjicharalambous et al. [34], who used 3D tagged MRI and a parameter sweep. The initial parameter set was chosen to match the Klotz relation. They found that the HO law provided the best balance between identifiability and model performance. Recently, Nasopoulou et al. [58] used 2D tagged MRI to improve the identifiability of parameters of the Guccione law by combining deformation and energy analysis to uniquely constrain the parameters. In more detail, the exponential scaling factor α was estimated through the minimization of an energy-based cost function. Then, a mechanical simulation was performed in a second step to optimize the linear isotropic parameter with a cost function based on differences in displacements. For this, the first frame and the two last frames of diastole were used to be compared to simulations of passive inflation. Finsberg et al. [20,21] used the backward displacement method to find the unloaded configuration and fitted one parameter of a reduced HO law with an initial guess of other parameters similar to Asner et al. [4].

The empirical EDPVR as proposed by Klotz et al. was utilized as a fitting target by, e.g. Nordsletten et al. [63], to estimate the unloaded geometry of human hearts. Simulations of passive inflation were conducted and compared to the Klotz relation, using least-square constraints on volume and pressure. In 2013, Krishnamurthy et al. [46] estimated the unloaded bi-ventricular geometry using a backward displacement method. They scaled parameters of a reduced HO law to fit the empirical Klotz curve by adjusting them manually and varying exponential parameters within a range of 15% of their initial values taken from experiments performed on canine tissue [17]. In 2018, Palit et al. [66] provided a summary of parameters of the HO law available in literature and examined the influence of ped, fiber orientations and geometry on the estimated parameters. They used an objective function which minimized the differences between the simulated and real LV cavity volume and used the Klotz curve as a fitting target albeit they had no pressure recordings and assumed ped to be 10 mmHg. The initial parameters were estimated by a Latin hypercube sampling (LHS) which generated 50 initial data sets. Sack et al. [72] used a similar approach, minimizing the error with respect to both pressure and volume. Initial parameters were fitted to data from [77] and scaled with one scaling parameter for the linear terms and one for the exponential terms to match the Klotz relation. For the passive filling calibration, they defined an objective function as the difference in pressure values along the PV-curve combined with a single measure of end diastolic volume, Ved. Genet et al. [26] calibrated two parameters of the Guccione law [32] using two nested loops: first, to optimize a parameter b, that defines the nonlinearity of the stress-strain relationship, such that the resulting loading curve is close to the Klotz curve and second, the scaling parameter of the Guccione law was optimized for a given b such that computed and prescribed end-diastolic pressures match.

In this paper, we present a novel model function-based fitting method (MFF) to find personalized material parameters for passive mechanical modeling of the heart. Only image data at one time point to create the anatomical model and one measured data point of the EDPVR are required as an input. The approach simultaneously personalizes passive material parameters and generates an unloaded reference configuration which is crucial for the image-based modeling of biomechanical LV diastolic function. In this regard, we also present an improved backward displacement algorithm where robustness is enhanced by a novel line search strategy. The MFF method was tested on a cohort of 19 LV meshes [49]. Excellent agreement with the empirical Klotz EDPVR was obtained for all cases under study. As the determination of an unloaded reference configuration and the identification of parameters is carried out simultaneously, the method is highly efficient, requiring only a small number of forward simulations (≤ 10 for all cases). Hence, computational costs were only about 2 to 3 times the cost of a standard passive inflation experiment. The versatile workflow is applicable to a large variety of constitutive models, model functions, and FE formulations and can be coupled to established FE software packages with relative ease. A thorough sensitivity analysis demonstrates the robustness of the method with regard to input uncertainty.

## 2 Methods

### 2.1 Patient data

Clinical data from the CARDIOPROOF study (NCT02591940), a recent clinical trial, was available, which includes data of *N*_AS_ = 12 aortic valve stenosis (AS) patients and *N*_CoA_ = 7 aortic coarctation (CoA) patients. As indicator for treatment, valve area and/or systolic pressure drop across the valve was taken into account for AS cases, whereas for CoA cases treatment indicators included an echocardiographically measured peak systolic pressure gradient across the stenotic region greater than 20 mmHg (2.66 kPa) and/or arterial hypertension. The institutional Research Ethics Committee approved the study following the ethical guidelines of the 1975 Declaration of Helsinki. From the participants' guardians written informed consent was attained. A detailed description of the data acquisition process and clinical protocols used in this study were reported in [19]. Pressure measurement in the LV, namely invasive catheterization, was routinely acquired in patients suffering from CoA only, making measured values of $p_{\text{ed}}^{\text{dat}}$ solely available for this group. For AS cases $p_{\text{ed}}^{\text{dat}}$ was determined empirically by statistically analyzing a reference data pool of *N* = 290 patient cases treated for AS. For more details on the reference data pool see the supplementary material of [49].

### 2.2 Unloaded reference configuration

**Fig. 1:**
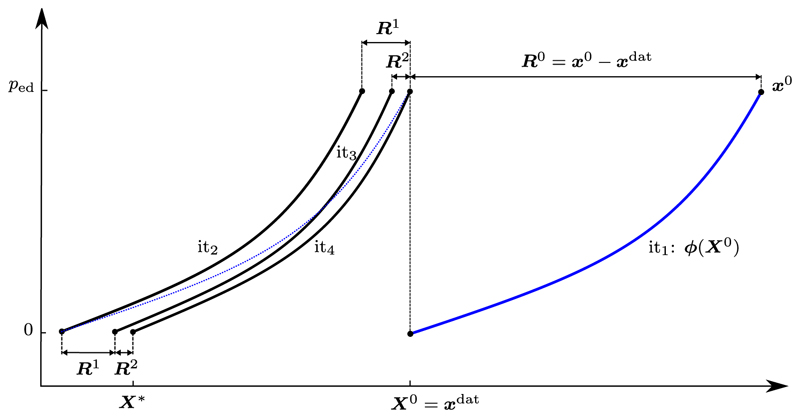
Schematic for Sellier's backward displacement method, see Algorithm 3 in Appendix A. A forward inflation problem *ϕ* (blue curve) is solved in the first iteration (it_1_), where a measured *in vivo* pressure *p*_ed_ is applied to an interim reference configuration with coordinates ***X***^0^ = ***x***^dat^. This generates an updated configuration with coordinates ***x***^0^. Subsequently, the per node displacement vector between the updated deformed configuration and the target *in vivo* configuration (***R***^0^) is computed. Finally, the reference configuration is updated by subtracting this per node displacement vector (blue dashed line). This iterative procedure is repeated until a given error tolerance *∈* between the computed reference configuration and the given *in vivo* configuration is reached. Here, the algorithm converges to the unloaded reference configuration X* after four iterations. (For interpretation of the colors in the figure(s), the reader is referred to the web version of this article.)

To find an unloaded reference configuration Sellier [75] proposed an iterative, fixed-point method for general elasto-static problems, see Fig. 1 for a schematic and Algorithm 3 in Appendix A. In each iteration *k* a forward problem ***ϕ***(***X**^k^*) is solved, where a measured *in vivo* pressure is applied to an interim reference configuration with coordinates ***X**^k^*. This results in updated configurations with coordinates ***x**^k^* and, subsequently, the per node displacement vector between the updated deformed configuration and the target *in vivo* configuration ***R**^k^* is computed. Finally, the reference configuration is updated by subtracting this per node displacement vector. This iterative procedure is repeated until a given error tolerance *∈* between the computed reference configuration and the given *in vivo* configuration is reached and hence the unloaded reference configuration X^*^ is found. Rausch et al. [68] augmented this approach, see Algorithm 4 in Appendix A, based on Aitken's delta-squared process [1] that increases the convergence rate and also significantly accelerates the method. Both fixed-point approaches are simple and versatile and can be coupled to existing FE software packages with relative ease and have thus been applied to a number of biomechanical modeling problems, e.g., [41,43,87].

However, for some of our LV models the augmented approach still diverged and unfavorable updates of the reference vector even resulted in a failure of the algorithm. Since our parameter fitting approach described later in Section 2.3.2 is based on a robust unloading strategy for soft tissues, we further improved this iterative method by a line search strategy based on Armijo [3], see Algorithm 1. Here, unfavorable search directions are damped and with this improvement the unloading algorithm was robust for all cases and in most cases the procedure was sped up. See Table A.10 for a comparison of the robustness and computational times for the three specified unloading algorithms.

We successfully applied Algorithm 1 to all 19 LV models of the cohort from Marx et al. [49], as well as meshes of bi-ventricular, see Karabelas et al. [42], and four-chamber models from the cohort presented by Strocchi et al. [78].

### 2.3 Constitutive fitting

#### 2.3.1 Constitutive models

The myocardium is considered as a non-linear, hyperelastic, nearly incompressible, and orthotropic material with a layered organization of myocytes and collagen fibers that is characterized by a right-handed orthonormal set of basis vectors; see [10] for a great review on material properties and constitutive modeling of the passive mechanical behavior of myocardial tissue. The basis vectors consist of the fiber axis ***f***_0_, which coincides with the orientation of the myocytes, the sheet axis ***s***_0_ and the sheet-normal axis ***n***_0_. To enforce the condition of a nearly incompressible material, the strain energy function ψ, is split into a volumetric *U*(*J*) and an isochoric part ψ_isc_(***C***):

(1) $$\Psi \left(\text{C}\right)=U\left(J\right)+\Psi_{\text{isc}}\left(\text{C}\right).$$

In this relation, ***C*** = ***F***^Τ^***F*** is the right Cauchy–Green deformation tensor, with ***F*** the deformation gradient. *U* (*J*) is composed of the bulk modulus *κ* ≫ 0 *kPa* and a penalty term related to the Jacobian of the deformation gradient *J* = det(***F***) and we choose

(2) $$U\left(J\right)=\frac{\kappa }{2}\text{In}{\left(J\right)}^{2}$$

for all considered material models. One of the simplest constitutive laws to model rubber-like materials is the isotropic *Demiray model* [16]

(3) $$\Psi \left(\text{C}\right)=U\left(J\right)+\frac{a}{2b}\left\{\text{exp}\left[b\left({\bar{I}}_{1}-3\right)\right]-1\right\},$$

with the invariant ${\bar{I}}_{1}=tr(\bar{\text{C}})$ and parameters *a* > 0 *kPa* and *b* > 0. Here, $\bar{\text{C}}:=J^{-2/3}\text{C}$ is the isochoric part of the right Cauchy–Green tensor resulting from the multiplicative split proposed by Flory [22] to model the nearly incompressible behavior of elastic materials.

Algorithm 1 **Augmented Sellier's Inverse Method with Armijo strategy.**

1: initialize ***X***^0^ = ***x***^dat^; ***R***^0^ = 0; *k* = 0; *β* = 1.0

2: initialize *l*_min_ which determines the maximal number of Armijo steps; we used $l_{\min}=\frac{1}{8}$

3: **do**

4:   $\text{Λ}=\left\{1,\frac{1}{2},\frac{1}{4},\ldots ,{𝓁}_{\min}\right\}$

5:   **for** *l* ∈ Λ **do**

6:     solve forward problem, ***x**^l^* = *ϕ* (***X**k*)

7:     calculate nodal error vector, ***R**^l^* = ***x**^l^* = ***x**^dat^*

8:     **if** *k* > 0 **then**

9:       update augmentation parameter,$\beta =-\beta \frac{R^{k-1}:\left[R^{𝓁}-R^{k-1}\right]}{\left[R^{𝓁}-R^{k-1}\right]:\left[R^{𝓁}-R^{k-1}\right]}$

10:     **end if**

11:     update reference vector, ***X**^l^* = ***X**^k^* = *lβ**R**^l^*

12:     compute maximal nodal error, $r^{𝓁,}\left\|𝒙\right\|\infty =\underset{i\in \left[1,N_{\text{nodes}}\right]}{\max}{\left\|{𝒙}_{i}^{𝓁}-{𝒙}_{i}^{\text{dat}}\right\|}_{2}$

13:     **if** *k* = 0 **or** $r^{𝓁,∥𝒙∥\infty }<r^{∥𝒙∥\infty }$ **break**

14:   **end for**

15:   determine l* such that $∥{𝑹}^{𝓁*}∥=\underset{\text{𝓁}\epsilon \Lambda }{\min}\left\{∥{𝑹}^{𝓁}∥\right\}$

16:   update, ***x**^k^* = **x**^l*^, ***R**^k^* = **R**^l*^, ***X***^*k*+1^ = **X**^l*^

17:   compute maximal nodal error, $r^{∥𝒙∥\infty }=\underset{i\in \left[1,\;N_{\text{nodes}}\right]}{\max}∥{𝒙}_{i}^{k}-{𝒙}_{i}^{\text{dat}}{∥}_{2}$

18:   update counter, *k* = *k* + 1

19: **while** $r^{∥𝒙∥\infty }\geq \epsilon$

20: unloaded reference configuration, X* = ***X**^k^*

*Single Fung-type exponential models*, e.g., Guccione et al. [31], Usyk et al. [82], can be generalized to

(4) $$\Psi \left(𝑪\right)=U\left(J\right)+\frac{a}{2}\left[\text{exp}\left(\bar{Q}\right)-1\right],$$

where

(5) $$\bar{Q}=b_{\text{ff}}{\bar{E}}_{\text{ff}}^{2}+b_{\text{ss}}{\bar{E}}_{\text{ss}}^{2}+b_{\text{nn}}{\bar{E}}_{\text{nn}}^{\text{2}}+2b_{\text{fs}}{\bar{E}}_{\text{fs}}^{\text{2}}+2b_{\text{fn}}{\bar{E}}_{\text{fn}}^{\text{2}}+2b_{\text{ns}}{\bar{E}}_{\text{ns}}^{\text{2}}.$$

Here,

$${\bar{E}}_{\text{ff}}={\text{f}}_{0}\cdot \bar{\text{E}}{\text{f}}_{0},\;{\bar{E}}_{\text{ss}}={\text{S}}_{0}\cdot \bar{\text{E}}{\text{S}}_{0},\;{\bar{E}}_{\text{nn}}={\text{n}}_{0}\cdot \bar{\text{E}}{\text{n}}_{0},$$

$${\bar{E}}_{\text{fs}}={\text{f}}_{0}\cdot \bar{\text{E}}{\text{s}}_{0},\;{\bar{E}}_{\text{fn}}={\text{f}}_{0}\cdot \bar{\text{E}}{\text{n}}_{0},\;{\bar{E}}_{\text{ns}}={\text{n}}_{0}\cdot \bar{\text{E}}{\text{s}}_{0}$$

are projections of the Green-Lagrange strain tensor $\bar{\text{E}}=\frac{1}{2}\left(\bar{\text{C}}-\text{I}\right)$ and parameter *a* > 0 *kPa* serves as a stress-like scaling. The dimensionless parameters *b*_ff_, *b*_ss_, *b*_nn_ > 0 account for the mechanical behavior of the tissue along the fiber (f), sheet (s) and sheet-normal (n) direction, and *b*_fs_, *b*_fn_, *b*_ns_ > 0 account for structural interactions. For parameter values of the different single Fung-type exponential models considered, see Table 1.

Holzapfel and Ogden [39] proposed a *separated Fung-type exponential model* which can be generalized to

(6) $$\Psi \left(𝑪\right)=U\left(J\right)+\frac{a}{2b}\left\{\text{exp}\left[b\left({\bar{I}}_{1}-3\right)\right]-1\right\}+\sum_{\text{i=f,s,n}}\frac{a_{i}}{2b_{i}}\left\{\text{exp}\left[b_{i}{\left(I_{4i}-1\right)}^{2}\right]-1\right\}+\sum_{\text{i=fs,fn,sn}}\frac{a_{ij}}{2b_{ij}}\left\{\text{exp}\left[b_{ij}{\left(I_{8ij}\right)}^{2}\right]-1\right\},$$

with parameters *a*_(•)_ ≥ 0 *kPa* and *b*_(•)_ > 0, invariant ${\bar{I}}_{1}=\text{tr}(\bar{\text{C}})$, unimodular fourth-invariants

$$I_{4\text{f}}=\max\left({\text{f}}_{0}\cdot \text{C}{\text{f}}_{0},1\right),\;I_{\text{4s}}=\max\left({\text{s}}_{0}\cdot \text{C}{\text{s}}_{0},1\right),\;I_{4\text{n}}=\max\left({\text{n}}_{0}\cdot \text{C}{\text{n}}_{0},1\right),$$

such that contributions of compressed fibers are excluded, and interaction-invariants

$$I_{\text{8fs}}={\text{f}}_{0}\cdot \text{C}{\text{s}}_{0},\;I_{\text{8fn}}={\text{f}}_{0}\cdot \text{C}{\text{n}}_{0},\;I_{\text{8sn}}={\text{s}}_{0}\cdot \text{C}{\text{n}}_{0}.$$

Note that for the anisotropic contributions in Equation (6) the deformation gradient ***F*** and the right Cauchy-Green tensor ***C*** remain unsplit to avoid nonphysical results [35,74].

Comparing to experimental data, Holzapfel and Ogden reduced the constitutive law with the full set of invariants (6) to a simplified model with the parameters *a*, *a*_f_, *a*_s_, *a*_fs_ > 0 *kPa* and *b*, *b*_f_, *b*_s_, *b*_fs_ > 0.

Further, several papers [18,33,77] deal with in-plane and out-of plane dispersion of collagen fibers along the ***f***_0_ and ***s***0 direction. According to Eriksson et al. [18] this is modeled by modifying the unimodular fourth-invariants to

(7) $$I_{4i}^{*}=\kappa_{i}{\bar{I}}_{1}+\left(1-3\kappa_{i}\right)I_{4i},\;i\in \text{f},\text{s}.$$

Dispersion parameters have been identified previously by mechanical experiments on passive cardiac tissue by Sommer et al. [77] and are set to *K*_f_ = 0.08 and *K*_s_ = 0.09.

For parameter values of the considered separated Fung-type exponential models, also often referred to as HO-type models, see Table 2.

#### 2.3.2 Fitting with model function

Due to limited availability of clinical data representing the EDPVR, the passive biomechanical material model is fitted to the empirical Klotz EDPVR estimated from a single measured PV-pair [45]. A more detailed description about the method proposed by Klotz et al. [45] can be found in Appendix B.

Fitting is done by adjusting material parameters of the constitutive relation during the unloading procedure (Algorithm 1) such that the cavity volume of the unloaded reference configuration X* matches $V_{\text{0}}^{\text{klotz}},$, see Equation (B.1), and such that the simulated PV-curve matches the EDPVR as predicted by Equation (B.2). In particular, the parameters are varied in each unloading step as follows: consider

(8) $$\text{Φ}\left(𝒙,{𝒙}_{0}\right)=\frac{a}{2b}\left\{\text{exp}\left[b\left(\frac{𝒙-{𝒙}_{0}}{{𝒙}_{0}}\right)\right]-1\right\},$$

a function commonly used to describe the passive diastolic PV relation [61], with parameters *a* and *b*. We use a Levenberg-Marquardt least-squares algorithm to fit the model function Φ(*x*, *x*_0_) to the Klotz relation, where $\chi \in \left\{V_{i}^{\text{klotz}}\left(p_{i}\right):i=1,\ldots ,n_{ls}\right\}$ are the volumes as predicted by the Klotz power law (B.2) and *x*_0_ is the predicted volume of the unloaded geometry $V_{\text{0}}^{\text{klotz}},$ (B.1). Here, *n*_ls_ is the number of loading steps and *p_i_* are equidistant pressures in the interval [0, *p*_ed_], hence, *p*_0_ = 0 and *p*_*n*_ls__ = *p*_ed_. After convergence of the fitting method we get the parameter set $\left[a_{\text{fit}}^{\text{klotz}},b_{\text{fit}}^{\text{klotz}}\right].$. Both fitting parameters are always strictly positive, due to the convexity of the Klotz relation and the design of the model function (8). In a second step the procedure is repeated for the simulated PV-curve in the current step *k* of the unloading algorithm with $\chi \in \left\{V_{i}^{\text{sim}}\left(p_{i}\right):i=1,\ldots ,n_{ls}\right\}$ being the volumes at the different loading points and *x*_0_ the cavitary volume of the current reference configuration ***X**^k^*, to obtain the parameter set $\left[a_{\text{fit}}^{\text{sim}},b_{\text{fit}}^{\text{sim}}\right].$. The parameter fitting is an extension of the unloading algorithm described above, see also Algorithm 2. Thus, we use the same index *k* to specify the current iteration step while index *i* specifies the current loading step of the forward simulation. Note that $a_{\text{fit}}^{\text{sim}}>0$ holds, due to the fact that an increase in pressure leads to an increase in volume, and $b_{\text{fit}}^{\text{sim}}\neq 0,$ due to the design of the model function (8). We compute the scalings

(9) $$a^{\text{scale}}=\frac{a_{\text{fit}}^{\text{klotz}}}{a_{\text{fit}}^{\text{sim}}},\;b^{scale}=\frac{b_{\text{fit}}^{\text{klotz}}}{\left|b_{\text{fit}}^{\text{sim}}\right|}.$$

Here, the absolute value of $b_{\text{fit}}^{\text{sim}}$ is chosen, as this fitting parameter might be negative in rare circumstances when the simulated PV-curve is concave. Also, for the unlikely case of a linear PV-curve, $b_{\text{fit}}^{\text{sim}}$ is close to 0, leading to a very large scaling parameter *b*^scale^. Hence, to avoid large jumps in the material parameters, we define the bounding interval $ℐ=\left[\frac{1}{5},5\right]$ with *a*^scale^ and *b*^scale^ taking the value of the closest bound if *a*^scale^ ∉ *ℐ* or *b*^scale^ ∉ *ℐ*, respectively.

Finally, all parameters of the material model in Equations (3) to (6) are updated for the upcoming unloading step *k* + 1 according to

(10) $$a_{\left(•\right)}^{k+1}=a^{\text{scale}}\cdot a_{\left(•\right)}^{k},\;b_{\left(•\right)}^{k+1}=b^{\text{scale}}\cdot b_{\left(•\right)}^{k}$$

**Fig. 2:**
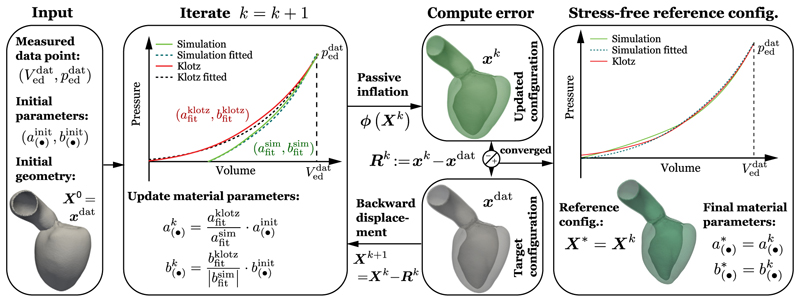
Graphical representation of the fixed-point algorithm to identify myocardial passive material parameters and the unloaded reference configuration. Starting from an initial geometry ***X***^0^ obtained from image data and initial parameters $a_{\left(\cdot \right)}^{\text{init}},b_{\left(\cdot \right)}^{\text{init}}$ from the literature, we perform an iterative procedure to find estimates for the unloaded reference configuration X* and personalized passive material parameters $a_{\left(\cdot \right)}^{*},b_{\left(\cdot \right)}^{*}$. While iterating, the simulated loading curve (in green) gets closer to the empiric EDPVR [45] (in red) which is determined by a measured pressure volume pair ($p_{\text{ed}}^{\text{dat}}$, $V_{\text{ed}}^{\text{dat}}$). Convergence of the algorithm is based on error estimates given in Section 2.3.3.

The aim of this scaling step is to get the current simulated loading curve to converge against the empiric EDPVR. When the material is too stiff $\left(V_{0}^{\text{klotz}}<V_{0}^{\text{sim}}\right)$, then stress-like *a*_(•)_. parameters are scaled with a value less than one (*a*^scale^ < 1) to soften the material; vice versa when the material is too soft $\left(V_{0}^{\text{klotz}}>V_{0}^{\text{sim}}\right)$, then the stress-like *a*_(•)_. parameters are scaled with a value greater than one (*a*^scale^ > 1) to increase stiffness. Further, when the simulated loading curve is too straight, parameters *b*_(•)_ are increased (*b*^scale^ > 1); and vice-versa when the simulated loading curve is too bent, then *b*_(•)_ are decreased (*b*^scale^ < 1). These changes to the current parameter setting are ensured by the independent fitting of the model function first to the Klotz-curve and second to the current loading curve and the scaling as described in Equations (9) and (10). Interactions between simultaneous changes of *a*_(•)_ and *b*_(•)_ parameters are settled with the iterative approach extending the unloading algorithm as described above. Upon convergence of the algorithm in step *k*, see Section 2.3.3 for convergence criteria, we get the unloaded configuration X* = ***X**^k^* and final parameter set $a_{\left(•\right)}^{*}=a_{\left(•\right)}^{k}\;\text{and}\;b_{\left(•\right)}^{*}=b_{\left(•\right)}^{k}$, see Algorithm 2 and Fig. 2.

Note that all discussed material models require that $a_{\left(•\right)}^{k}>0\;\text{and}\;b_{\left(•\right)}^{k}>0$ for all *k* and hence *a*^scale^ and *b*^scale^ have to be positive, which is guaranteed by design. Additionally, we point out that for all simulations where the initial loading function was concave the presented algorithm still converged in a satisfactory manner.

To verify the proposed MFF approach, a more costly cost function based fitting (CFF) approach was established and applied to the available patient data. For details see Section 3.4.

#### 2.3.3 Error estimates and goodness of fit

To check for convergence, detect potential stagnation, and measure goodness of fit of the proposed method, several error estimates were introduced, which are described in detail below. The difference of *V*_0_ and *V*_ed_ between the simulated and the empirical Klotz EDPVR was quantified by the difference of the initial volumes

(11) $$r^{V_{0}}=\left|V_{0}^{\text{klotz}}-V_{0}^{\text{sim}}\right|$$

where $V_{0}^{\text{sim}}$ is the cavity volume of the reference configuration ***X**^k^* at the current unloading step *k*, and the difference of end-diastolic volumes

(12) $$r^{\text{ed}}=\left|V_{\text{ed}}^{\text{dat}}-V_{\text{ed}}^{\text{sim}}\right|$$

where $V_{\text{ed}}^{\text{dat}}$ is the measured end-diastolic volume and $V_{\text{ed}}^{\text{sim}}$ is the cavitary volume of the inflated configuration ***x**^k^*. For convergence we chose an error tolerance of *∈*^vo1^ = 0.5% of $V_{\text{ed}}^{\text{dat}}$ such that *r*^ed^ < *∈*^vo1^ and *r*^*V*_0_^ < *∈*^vo1^.

The difference between the simulated and measured end-diastolic geometry was calculated by consideration of the maximal nodal error

(13) $$r^{{\left\|𝒙\right\|}_{\infty }}=\underset{i\in \left[1,N_{\text{nodes}}\right]}{\max}{\left\|{𝒙}_{i}^{k}-{𝒙}_{i}^{\text{dat}}\right\|}_{2},$$

where ||•||_2_ stands for the *l*_2_-norm and *N*_nodes_ represents the total number of nodes in the geometry. To obtain convergence, *r*^||*x*||^∞ was required to be smaller than 0.1 mm. Finally, we define the error in parameter update between unloading iterations as

(14) $$r^{\text{param}}=\max\left(\left|1-a^{\text{scale}}\right|,\left|1-b^{\text{scale}}\right|\right),$$

which is used to detect stagnation of the algorithm and required to be smaller than 0.001 for convergence.

To define the goodness of fit for the simulation outcome in terms of the fitted curve and the Klotz curve, the relative difference of initial volumes

(16) $$r^{V_{0},\text{rel}}=\frac{\left|V_{\text{0}}^{\text{klotz}}-V_{\text{0}}^{\text{sim}}\right|}{V_{\text{ed}}^{\text{dat}}}.10^{2},$$

and the relative area difference

(16) $$r^{A_{n},\text{rel}}=\frac{r^{A_{n}}}{A_{\text{koltz}}}.10^{2}$$

of the normalized curve to the area under the Klotz curve *A*_klotz_ were calculated. The absolute area difference *r*^*A_n_*^ between two curves in the 2D space is computed by the method introduced by Jekel et al. [40], which positions quadrilaterals *q* between two curves and subsequently calculates the sum of their areas

$$r^{A_{n}}=\sum_{q}A_{q},$$

using Gauss' area formula

$$A_{q}=\frac{1}{2}\left|x_{1}y_{2}+x_{2}y_{3}+x_{3}y_{4}+x_{4}y_{1}-x_{2}y_{1}+x_{3}y_{2}+x_{4}y_{3}+x_{1}y_{4}\right|,$$

where (*x_i_*, *y_i_*) represent the vertices of a quadrilateral.

Algorithm 2 **Fixed-point algorithm to identify myocardial passive material parameters and the unloaded reference configuration**

1: initialize geometry, ***X***^0^ = ***x***^dat^; *k* = 0; *β* = 1.0

2: initialize PV-pair from measured data $p_{\text{ed}}^{\text{dat}},V_{\text{ed}}^{\text{dat}}$

3: initialize default parameters with values from the literature, $a_{\left(•\right)}^{0}=a_{\left(•\right)}^{\text{init}}\;\text{and}\;b_{\left(•\right)}^{0}=b_{\left(•\right)}^{\text{init}}$

4: compute empirical EDPVR as proposed by Klotz et al. [45], see Appendix B, using PV-pair $p_{\text{ed}}^{\text{dat}},V_{\text{ed}}^{\text{dat}}$

5: fit model function Equation (8) to the Klotz relation and obtain $a_{\text{fit}}^{\text{klotz}}\;\text{and}\;b_{\text{fit}}^{\text{klotz}}$

6: **do**

7:     perform inverse method step, Algorithm 1 states 3–16, using parameters $a_{\left(•\right)}^{k},b_{\left(•\right)}^{k}$

8:     retrieve current reference configuration ***X***^*k*+1^ and simulated loading curve

9:     fit model function Equation (8) to current loading curve and obtain $a_{\text{fit}}^{\text{sim}}\;\text{and}\;b_{\text{fit}}^{\text{sim}}$

10:     compute scalings $a^{\text{scale}}=a_{\text{fit}}^{\text{klotz}}/a_{\text{fit}}^{\text{sim}}\;\text{and}\;b^{\text{scale}}=b_{\text{fit}}^{\text{klotz}}/\left|b_{\text{fit}}^{\text{sim}}\right|$

11:     update parameters $a_{\left(•\right)}^{k+1}=a^{\text{scale}}\cdot a_{\left(•\right)}^{k}\;\text{and}\;b_{\left(•\right)}^{k+1}=b^{\text{scale}}\cdot b_{\left(•\right)}^{k}$

12:     update counter, *k* = *k* + 1

13:     compute error estimates as in Section 2.3.3 and check convergence

14: **while** not converged

15: unloaded reference configuration: X* = ***X**^k^*

16: fitted parameter set: $a_{\left(•\right)}^{*}=a_{\left(•\right)}^{k}\;\text{and}\;b_{\left(•\right)}^{*}=b_{\left(•\right)}^{k}$

### 2.4 Numerical framework

The unloading and parameter estimation scheme was implemented in the FE framework Cardiac Arrhythmia Research Package (CARP) [83]. The simulations were constrained by applying spring-like boundary conditions at the rim of the clipped aorta and over the epicardial surface at the apex of the LV, respectively, see [49,79]. For all the passive inflation simulations we rely on solver methods established previously [9] which have been verified in a N-version benchmark study [47]. In brief, the linear systems were solved by a generalized minimal residual method (GMRES) with a relative error reduction of *∈* = 10^–8^. Efficient preconditioning was based on the library *PETSc* (https://www.mcs.anl.gov/petsc/). For all cases we used 100 loading steps to ramp up the pressure from *p* = 0 to *p* = *p*_ed_. To speed up computations, we limited the number of Newton steps to two for the passive inflation simulations during the unloading scheme. This did not alter simulation results and is justified as we were only estimating the unloaded reference configuration and material parameter sets. For the MFF with the Levenberg-Marquardt least-squares algorithm we used Python V. 3.8 and packages NumPy V. 1.16.6 and SciPy V. 1.2.3; see [55] for details on the implementation of this algorithm.

To validate our results, we performed a passive inflation experiment that starts with the found unloaded reference configuration and the determined parameter set. For this passive inflation we used a fully converging Newton algorithm with a relative *l*_2_-norm error reduction of the residual of *∈* = 10^−6^ and 100 loading steps. All goodness of fit measurements in the sections below were calculated using the PV-curve of this validation experiment.

## 3 Results

### 3.1 LV patient cohort results

**Fig. 3:**
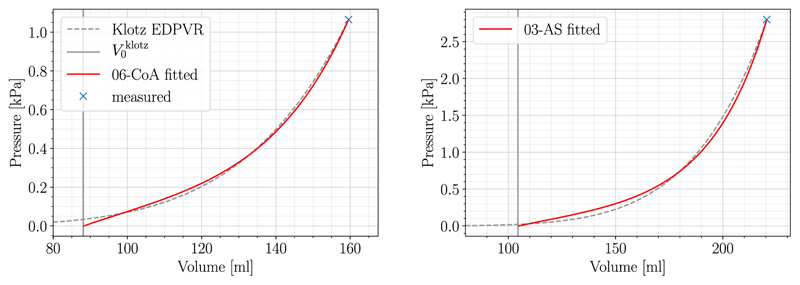
Fitting results of case 06-CoA (left) and 03-AS (right) are shown. The measured end-diastolic PV-point is marked in blue and used as a starting point for the Klotz EDPVR (dashed gray). $V_{\text{0}}^{\text{klotz}},$ is shown as a vertical line in solid gray, while the fitted relation is visualized in solid red.

The MFF method as described in Section 2.3.2 was executed for all *N* = 19 patient cases. Parameters of the reduced HO constitutive law were fitted using initial values from the literature, see Table 2. P1-P0-elements with the bulk modulus *k* = 650 *kPa* were utilized for all cases and simulations were run on the Vienna Scientific Cluster 4 (VSC-4) using 96 cores. Given the convergence criteria as presented in Section 2.3.3, the MFF algorithm converged for all 19 cases and fitting results are summarized in Table 3. Cases with best (06-CoA) and worst (03-AS) fit were distinguished by calculating the relative area difference *r*^*A_n_*,rel^, see Equation (16), and used for further analysis in the sections below. The Klotz curve and the simulated PV-curve for these two cases are visualized in Fig. 3. Even for the worst case the fitted model approximated the Klotz curve remarkably well, with an almost exact match in terms of $V_{\text{0}}^{\text{klotz}},$ and $V_{\text{ed}}^{\text{dat}}$. Indeed, the MFF method achieved excellent goodness of fit for all cases.

**Fig. 4:**
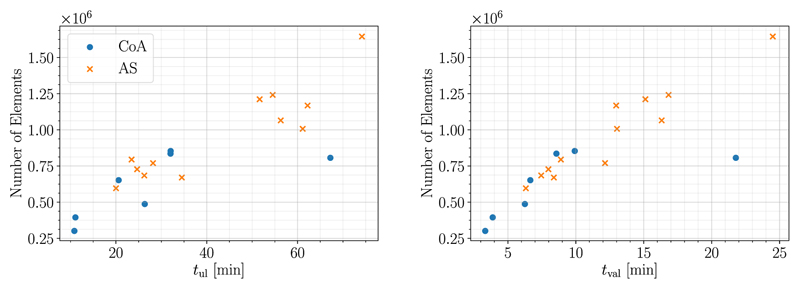
Relation of run-times for unloading *t*_ul_ (left) and validation *t*_val_ (right) to mesh size in terms of number of elements is visualized. Data from CoA Cases is shown as xxdjitsots, whereas data from AS Cases is marked as crosses.

Run-times *t*_ul_ for the MFF method which include unloading and parameter fitting were between 10.85 min and 74.18 min. For the subsequent passive inflation experiment for validation the run-times *t*_val_ were between 6.31 min and 24.50 min. As expected, computational cost increased with mesh size, see Fig. 4.

**Fig. 5:**
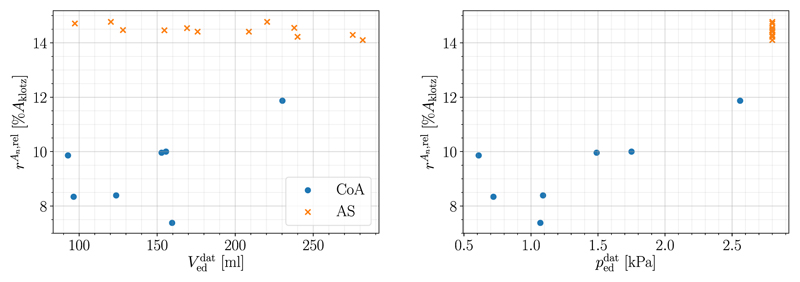
Influence of input parameters $V_{\text{ed}}^{\text{dat}}$ (left) and $p_{\text{ed}}^{\text{dat}}$ (right) on relative area difference *r*^*A_n_*,rel^ is shown. Data from CoA cases is marked as dots, whereas data from AS cases is marked as crosses.

Further, the influence of the input parameters $V_{\text{ed}}^{\text{dat}}$ and $p_{\text{ed}}^{\text{dat}}$ on goodness of fit, more precisely *r*^*A_n_*,rel^, is shown in Fig. 5. No trend can be recognized for different end-diastolic volumes $V_{\text{ed}}^{\text{dat}}$ while it appears that lower end-diastolic pressures $p_{\text{ed}}^{\text{dat}}$ might lead to better fits; this is investigated further in Section 3.3.

### 3.2 Results for different constitutive laws

The default constitutive law in Section 3.1, i.e., the reduced HO law with initial material parameters from Guan et al. [30], was chosen simply because this study is one of the most recent papers with fits to data from human ventricular myocardium. In this section, we show that the method described above works equally well for a large variety of constitutive laws, in fact most of the laws that are currently used to model passive myocardium.

**Fig. 6:**
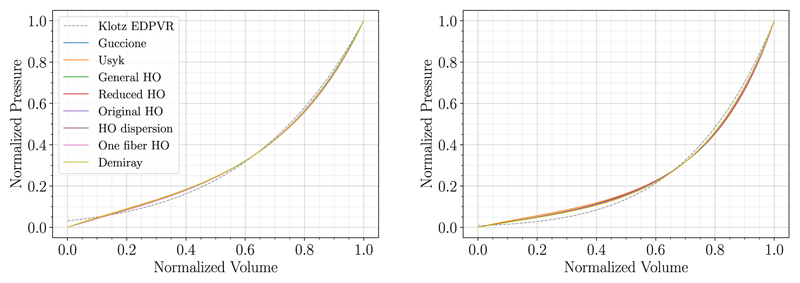
Fitting results of case 06-CoA (left) and 03-AS (right) using different constitutive laws are shown. The normalized Klotz EDPVR (dashed gray) is visualized along with the respective normalized fitted curves.

To this end, we applied the MFF method using the different constitutive laws listed in Tables 1 and 2 for the case with best (06-CoA) and the worst (03-AS) fit in Section 3.1. P1-P0-elements were used and all simulations were run on VSC4 using 96 cores. Normalized fitting results for cases 06-CoA and 03-AS are given in Fig. 6 and Table 4 showing excellent fits and almost similar PV-curves for all material laws. Due to its simplicity and isotropic nature, the Demiray material model resulted in the lowest number of iterations and shortest run-times for both example cases. More complex orthotropic models needed more iterations of the MFF algorithm and thus also run-times increased. Overall, MFF simulations were tractable with a computational run-time below 2.5 hours for all experiments.

#### 3.2.1 Results for locking-free finite elements

**Fig. 7:**
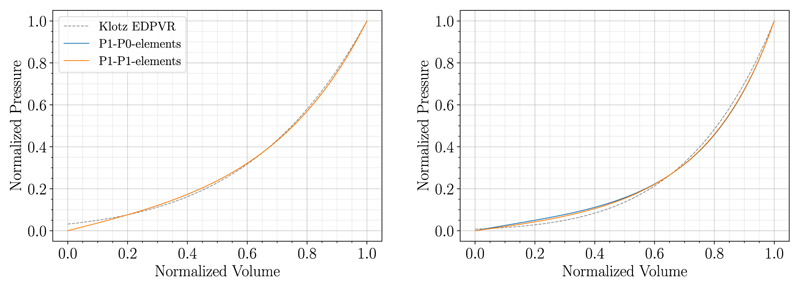
Fitting results using P1-P0-elements (solid blue) and locking-free P1-P1-elements (solid orange) are compared for case 06-CoA (left) and 03-AS (right). The Klotz EDPVR is shown in dashed gray.

It is well known that simple P1-P0-elements may suffer from locking effects and hence other FE formulations are required for certain applications where accurate stresses are essential. To show the capabilities of the MFF method in this scenario, we applied the algorithm to cases 06-CoA and 03-AS using stabilized, locking-free P1-P1-elements [44] and an incompressible material, i.e., 1/*k* = 0. Normalized fitting results for the two example cases are presented in relation to results obtained above using P1-P0-elements in Fig. 7 and Table 5. The PV-curves and the measures of goodness of fit *r*^*V*_0_,rel^ and *r*^*A_n_*,rel^ show that the method works equally well for stabilized P1-P1-elements. Looking at the parameter values in Table 5 we can see that *a*_scale_ is smaller and *b*_scale_ is larger for P1-P0-elements compared to the locking-free elements. This is anticipated as the fitting compensates for a certain degree of locking in the P1-P0-element formulation and thus it gives parameters that correspond to a softer material. Unsurprisingly, run-times were significantly longer when a stabilized P1-P1 FE formulation was used: 32 min vs 246 min for case 06-CoA and 61 min vs 364 min for case 03-AS, whereas iteration numbers of the MFF algorithm were similar.

We could achieve similar results with locking-free MINI elements as introduced by Karabelas et al. [44]; only run-times varied a bit compared to the stabilized P1-P1 formulation.

### 3.3 Sensitivity analysis

To show robustness of the proposed MFF methodology, a sensitivity analysis was performed to investigate the influence of clinical data uncertainty in terms of the end-diastolic PV-point used for computation of the Klotz EDPVR, changes in myocardial fiber orientation, altered model parameters used as initial guess and variations of the bulk modulus *k*, respectively. The sensitivity analysis was executed for example case 03-AS using the reduced HO material law and P1-P0-elements if not mentioned otherwise.

#### 3.3.1 Influence of variations in the end-diastolic PV-point on fitting outcome

**Fig. 8:**
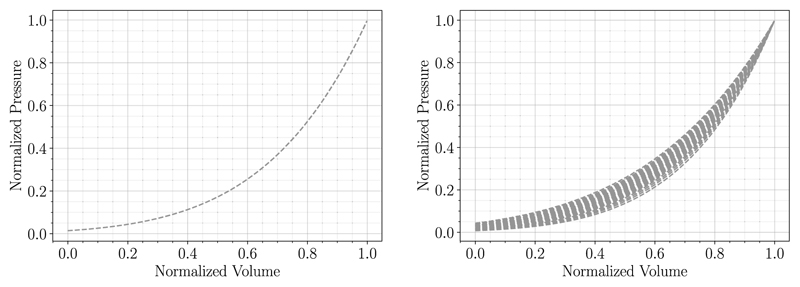
Behavior of the Klotz EDPVR relation is shown for varying end-diastolic PV-points. First, $V_{\text{ed}}^{\text{dat}}$ was varied while keeping $p_{\text{ed}}^{\text{dat}}$ constant and vice versa. Normalized curves match for alternating $V_{\text{ed}}^{\text{dat}}$ (left) while curves for alternating $p_{\text{ed}}^{\text{dat}}$ (right) differ in shape.

The behavior of the Klotz curve was investigated changing the inputs $V_{\text{ed}}^{\text{dat}}$ and $p_{\text{ed}}^{\text{dat}}$ independently and subsequently comparing the normalized curves. For that purpose, *n* = 20 evenly spaced values in the range of 93-282 mL for $p_{\text{ed}}^{\text{dat}}$ and 0.61-2.80 kPa for $p_{\text{ed}}^{\text{dat}}$ were chosen; range boundaries correspond to the respective minimum and maximum values in the patient cohort data. For the variable held constant the mean values (${\bar{V}}_{\text{ed}}^{\text{dat}}=\;175\;\text{mL}\;\text{and}\;{\bar{P}}_{\text{ed}}^{\text{dat}}=2.26\;\text{kPa}$) obtained from the cohort data were taken. First, $V_{\text{ed}}^{\text{dat}}$ was varied while keeping $p_{\text{ed}}^{\text{dat}}$ constant and vice versa. Normalized results of the generated curves are visualized in Fig. 8. It shows that curves generated with alternating $V_{\text{ed}}^{\text{dat}}$ match when normalized whereas normalized curves resulting from alternating $p_{\text{ed}}^{\text{dat}}$ differ.

**Fig. 9:**
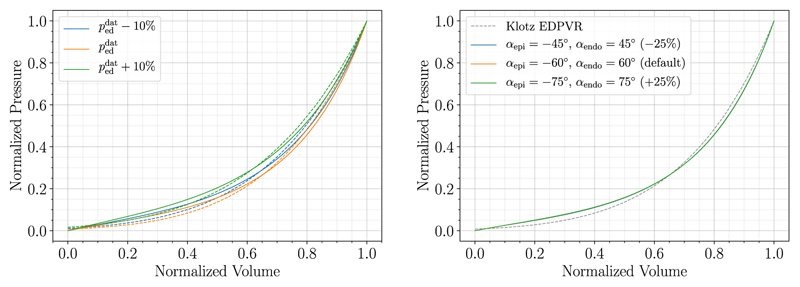
Fitting results with respective Klotz EDPVR (dashed curves) for case 03-AS are compared for varying $p_{\text{ed}}^{\text{dat}}$ (left) and changing fiber angles (right).

For that reason, varying $V_{\text{ed}}^{\text{dat}}$ will have little effect on the goodness of fit of the MFF method. We could also observe this behavior previously in Fig. 5. Hence, the sensitivity of the proposed MFF method was studied for varying $p_{\text{ed}}^{\text{dat}}$ only, considering deviations of ±10%. Results in Fig. 9 and Table 6 suggest that fitting results are better for Klotz EDPVR that have higher normalized pressure values, especially in the lower normalized volume region. Material parameters change for different values of $p_{\text{ed}}^{\text{dat}}$, but no particular trend is recognizable.

#### 3.3.2 Sensitivity to fiber orientation

Default myofiber orientations varying from −60° epicardial, *α*_epi_, to 60° endocardial, *α*_endo_, were chosen and perturbed by 25 %. This resulted in fiber angles of −75° and −45° in the outer wall and 75° and 45° in the inner wall, respectively. Sheet and sheet-normal directions were adapted accordingly to preserve the orthogonal system of local fiber coordinates. Results of fitting performance are visualized in Fig. 9 and values of fitted parameters and measures of goodness of fit are summarized in Table 7. As expected, parameter values are changing with the fiber orientation, as myocyte directions have a great impact on local material stiffness. Further, we can observe that the goodness of fit of the MFF algorithm is almost indifferent to changes in fiber orientation, leading to almost the same PV-curves in Fig. 9.

#### 3.3.3 Influence of initial model parameters on fitting outcome

To show the robustness of the method, the influence of variations in initial model parameters of the reduced HO material model was investigated. LHS from pyDOE V. 0.3.8 was used to create *n* = 10 different sets of initial scaling parameters {*a*_scale_, *b*_scale_} in the interval (0, 1). For all executed simulations the exact same parameter set and goodness of fit were obtained, also matching results with the default initial parameter set {*a*_scale_ = 1, *b*_scale_ = 1}, see case 03-AS in Table 3. While this shows the high robustness of the MFF algorithm, we cannot prove uniqueness and the run-times of the simulations varied significantly: *t*_ul_ = 39.48–302.25 min; depending on how close the initial parameters are to the final parameter set.

#### 3.3.4 Influence of variations in bulk modulus on fitting outcome

**Fig. 10:**
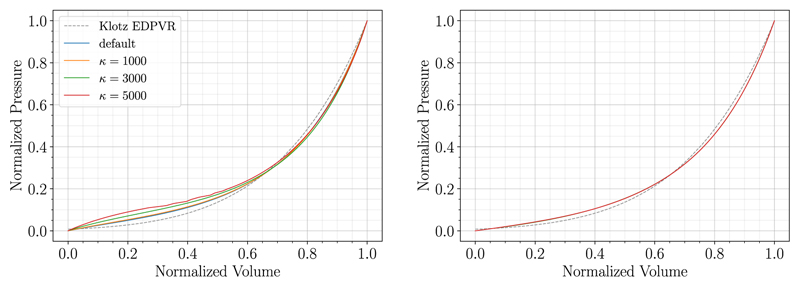
Fitting results for case 03-AS are compared for different values of *k* using P1-P0-elements (left) and stabilized P1-P1-elements (right).

Finally, sensitivity of the method to changes of the bulk modulus *κ* was studied using both P1-P0-elements and locking-free, stabilized P1-P1-elements. Simulations were run for example case 03-AS for *κ* = {1000, 3000, 5000} kPa. Additionally, default values were chosen for P1-P0-elements (*κ* = 650 kPa) and for P1-P1-elements (1/*κ* = 0). Results are visualized in Fig. 10 and summarized in Table 8. The forward simulations were not robust for P1-P0-elements and *κ* = 5000 kPa resulting in an irregular curve in Fig. 10(left). This is a shortcoming of the penalty formulation and P1-P0-elements, not the MFF method itself. We can see that for P1-P0-elements the material parameters vary greatly with the bulk modulus *κ*, compensating locking effects with material parameters that correspond to a softer material. However, when using P1-P1-elements, the method was not sensitive to changes in *κ*. Material parameters and goodness of fit are similar, which is a further indication of the robustness of the MFF algorithm.

### 3.4 Comparison to fitting with cost functional and parameter space sampling

To compare and verify the results of the MFF approach we implemented an *ad hoc* optimization procedure, based on cost functional fitting (CFF), to find sets of material parameters. To penalize deflections at lower and higher pressures and additionally penalize the difference to the volume of the unloaded geometry as determined by the Klotz relation (B.1), we chose the following cost functional:

(17) $$\begin{array}{l} J^{\text{so2}}=\frac{1}{2}{\sum_{p}\left(\frac{V^{\text{koltz}}\left(p\right)-V^{\text{sim}}\left(p\right)}{V_{\text{ed}}^{\text{dat}}}\right)}^{2}+\frac{1}{2}{\sum_{V}\left(\frac{p^{\text{koltz}}\left(V\right)-p^{\text{sim}}\left(V\right)}{p_{\text{ed}}^{\text{dat}}}\right)}^{2}\\ \;+\gamma {\left(\frac{V_{\text{0}}^{\text{klotz}}-V_{\text{0}}^{\text{sim}}}{V_{\text{ed}}^{\text{dat}}}\right)}^{2}. \end{array}$$

Here, the first term is the sum of squared normalized volume differences, the second term is the sum of squared normalized pressure differences, and the third term serves to further ensure that $V_{\text{0}}^{\text{klotz}},$ and $V_{\text{0}}^{\text{sim}},$ are close, with *γ* > 0 a weighting parameter; in all presented simulations we chose *γ* = 1. A downhill-Simplex based fitting (Nelder–Mead method) from SciPy was used to carry out the optimization, where we chose an absolute error of 0.001in the parameter update between iterations to be acceptable for convergence. We tried different varieties of CFF, the above described method with Equation (17) proved to be the most efficient.

*Results* The CFF approach was executed for the two example cases (06-CoA and 03-AS) with the reduced HO constitutive law, P1-P0-elements, and the bulk modulus *κ* = 650kPa. We used default values for the material parameters as in Table 2 scaled by a factor of 0.5 as we already expect from results in Table 3 that material parameters from the literature are in general too stiff; this scaling sped up computational times considerably.

Using the Nelder–Mead algorithm we optimized (i) *n*_opt_ = 1 scaling parameter (*a*_scale_) which was applied to all material parameters *a*_(•)_, whereas all *b*_(•)_ parameters of the model were scaled with 0.5; (ii) *n*_opt_ = 2 scaling parameters (*a*_scale_, *b*_scale_), with *a*_scale_ applied to all *a*_(•)_ parameters and *b*_scale_ applied to all *b*_(•)_ parameters of the model; and (iii) *n*_opt_ = 3 scaling parameters $\left(a_{\text{scale},}b_{\text{scale}}^{\text{iso}},b_{\text{scale}}^{\text{aniso}}\right),$ where *a*_scale_ is the scaling parameter that was applied to all *a*_(•)_ parameters; $b_{scale}^{iso}$ is the scaling parameter that was applied to *b* in the isotropic contribution of the constitutive law; and $b_{\text{scale}}^{\text{aniso}}$ is the scaling parameter that was applied to all *b*_(•)_ parameters of the anisotropic contribution.

We used bounds such that all scaling parameters are >0.1 to prevent that parts of the constitutive law are eliminated. The case *n*_opt_ = 4, where additionally afrom the isotropic contribution and all *a*_(•)_ from the anisotropic contribution were scaled by two different parameters, was not converging for the patient-specific cases within a reasonable amount of time (72 hours). We performed the optimization using values from the literature, see Table 2, as starting values (no init) as well as using the MFF approach to generate initial guesses for the parameter scalings (init).

**Fig. 11:**
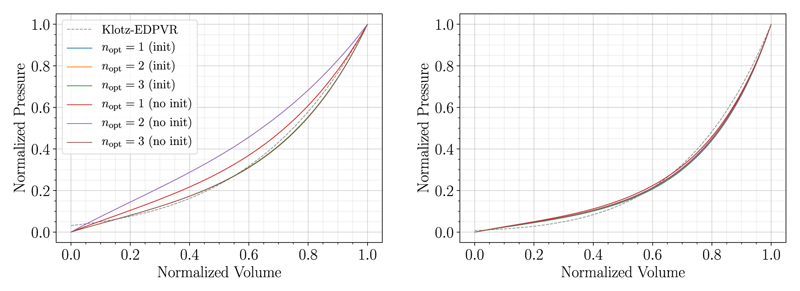
Normalized fitting results of case 06-CoA (left) and case 03-AS (right) executed using the reduced HO constitutive law with initial guesses from literature (no init) and with a MFF initialization step (init) as initial guess. Outcomes of the CFF method, optimizing one, two, or three parameters, *n*_opt_ = {1, 2, 3}, are compared.

Results are summarized in Table 9 and in Fig. 11 and we see that the worst fit was obtained for case 06-CoA optimizing two parameters and using initial values from the literature (no init), where the Nelder–Mead method got stuck in a local minumum. For *n*_opt_ = 1 similar results were reached when using values from the literature (no init) and initial scalings generated by a MFF (init). Best fits were acquired for *n*_opt_ = 2 with MFF initialization and *n*_opt_ = 3 in both variants. For case 03-AS obtained fitted parameters and goodness of fit were very similar for both initialization variants. Overall, results did not deviate much when one, two, or three parameters were optimized. In general, simulations run with default parameters from the literature needed more Nelder–Mead iterations compared to simulations with MFF iterations and thus also run-times were longer (>1.5 times as long).

**Fig. 12:**
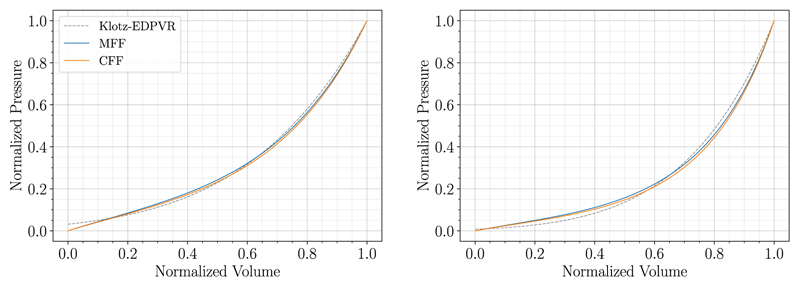
Comparison of fitting outcomes of MFF and CFF for case 06-CoA (left) and case 03-AS (right).

*Comparison of MFF and CFF approaches* Fitting outcomes of MFF (Table 3) and CFF (Table 9) agree well in terms of fitted parameters and measures of goodness of fit. Also the PV-curves of the MFF and CFF approach match almost exactly in Fig. 12 where the MFF result is compared to the best fit of the CFF method. However, since a full unloading step has to be solved in each Nelder-Mead iteration, the number of passive inflation simulations for the CFF approach is significantly larger compared to the MFF method: 79/156/516 vs 8 for 06-CoA and 110/460/835 vs 10 for 03-AS for CFF no init and *n*_opt_ = 1/2/3 fitted parameters vs. MFF; thus also the run-time of the CFF approach was considerably longer compared to the MFF approach: 330/546/2095 min vs 32 min for 06-CoA and 541/2197/3974 min vs 61 min for 03-AS for CFF no-init and *n*_opt_ = 1/2/3 fitted parameters vs. MFF.

## 4 Discussion

### 4.1 Comparison to other methods and the state-of-the-art

This study describes a novel methodology to fit passive mechanical parameters of the myocardium. Additionally, the algorithm computes an unloaded reference configuration where a new line search strategy was implemented that improves the robustness of a previously introduced backward-displacement method. We have demonstrated that the presented MFF method is an efficient, robust, and versatile approach for the automated identification of model parameters from clinical image data using the empirical EDPVR as proposed by Klotz et al. [45] as a fitting target. With this, the MFF algorithm only requires image or mesh data from one time instance during diastolic filling and a single measured data point of the EDPVR as inputs. Hence, the MFF method does not depend on a known approximation of the reference configuration which is a limitation of previous works [34,58]. This is of particular interest as often only a single anatomical snapshot taken within the diastatic window is available from clinical studies. Alternatively, previously generated geometries can be used for the MFF algorithm and thus no additional segmentation work is required to estimate material parameters for already existing meshes. Note that in many cases pressure and volume at end-diastole and image acquisition are not synchronized as both involve different modalities, e.g., catheter-based pressure measurement vs. echocardiography/CT/MRI. However, the presented method will still work if the image is scanned during early diastole or diastasis given an estimate of the pressure, *p*^image^, in the image-based geometry. Then, the first step of the unloading algorithm will be performed using *p*^image^ and the maximal nodal error comparing simulated and measured geometries Equation (13) is computed at this pressure. See also Finsberg et al. [21] for a similar workflow.

Contrary to other studies that jointly estimate material parameters and the unloaded reference configuration [4,21,20,62], we did not require to fix any parameters of the material law. To the best of our knowledge this is thus the first study that allows to fit not only for the predicted volume of the reference configuration *V*_0_ but also for the shape of the EDPVR. In opposition to studies above, we could apply our algorithm to much finer meshes (up to 1.5 million elements) and still keep simulations tractable with regard to computational cost. This is essential for many translational application of computational models in industry or in the clinic where time constraints apply.

Parameter estimation results are in line with previous studies where experimental parameters from the literature were also often considered as too stiff [41,72,84]. Fittings are in a similar range compared to [26, Table 1], [34, Figure 10], and [62, Table 2] for the Guccione law; and [4, Table 6] and [21, Table A1] for the one-fiber HO law; in these studies the authors were also able to reproduce the Klotz curve. Comparison of parameter values presented in other studies is difficult as these did not take the Klotz relation into account [58,90] or used different ratios between parameters [72].

To verify our novel MFF approach we implemented an *ad hoc* CFF based optimization procedure. Here, MFF and CFF approaches yield similar fitting results distinguishing the MFF approach as the superior method due to significantly shorter run-times and a higher robustness. While the MFF approach converged for all examined patient-cases, the more expensive CFF approach did not. This was due to time constraints on clusters and numerical problems stemming from instabilities of the forward solver owing to very soft material parameters chosen by the Nelder–Mead algorithm. For the latter case, we returned a very high cost, however, for some patient-specific geometries this still led to convergence problems. We further showed that even if a CFF approach is pursued, the MFF method can be used as a fast approach to generate an initial guess to improve convergence and efficiency. Note that for the CFF approach with *n*_opt_ = 3 scaling parameters, we fitted the material parameters of the isotropic and the anisotropic contribution independently. Nevertheless, we are aware that it is hardly possible to estimate the degree of anisotropy based solely on pressure-volume data. Here, experimental setups – as, e.g., described in papers mentioned above for default material parameters (Tables 1 and 2) – are by far better suited. For the MFF approach we thus keep the degree of anisotropy as it was given by default parameter sets and we do not distinguish between isotropic and anisotropic parts. For the sake of comparison, we tried the CFF approach also with 3 and 4 fitting parameters. As we can see in Table 9 the CFF approach with 3 fitting parameters did not improve results. Further, the CFF approach with 4 fitting parameters did not converge within a reasonable amount of time. In regard of the methods discussed in this paper we thus conclude that it is not worth pursuing an independent fitting of the isotropic and anisotropic contribution and, consequently, it is better to keep the degree of anisotropy as it was determined in experiments.

There are several other works that discuss the determination of the reference configuration from a loaded state, e.g., [29,48,67]. Each of these algorithms, as well as Algorithms 3 and 4, can be used instead of Algorithm 1 as a basis for the presented MFF approach to fit passive mechanical parameters of the myocardium. We only need the loading curve generated by inflating the unloaded reference configuration to a prescribed pressure as an input.

Finally, compared to some above-mentioned studies, the proposed method can be coupled with relative ease to established FE software packages. It only requires a limited number of passive inflation simulations, a standard experiment in all continuum mechanics simulators, and basic least-squares fitting tools which are available through open-source packages such as SciPy.

### 4.2 Computational costs

For all patient-specific cases, run-times for the MFF method (between 10.85 min and 74.18 min) were only 2 to 3 times higher compared to a standard passive-inflation experiment. Note that a further speed-up is possible by using only one Newton step during unloading, making the MFF method sometimes even faster than a single passive inflation experiment. However, in this case we observed stair-casing effects in the simulated PV-curve that might affect results to a minor degree. Also, a considerably lower number of loading steps will accelerate simulation times. In this scenario, the least-squares fitting to the piece-wise linear PV-curve could be less accurate.

The high efficiency of the parameter fitting is of utmost importance as the computational burden imposed by high-resolution patient-specific models demands fast numerical methods to keep simulations tractable.

### 4.3 Versatility of the workflow with respect to material laws and FE formulations

The MFF approach works for various material models including the most widely used passive cardiac tissue models, e.g., HO-type and Fung-type materials as introduced in Section 3.2. Run-times and iteration numbers of the MFF algorithm varied to a small degree with complexity and anisotropy of the model. Convergence with criteria discussed in Section 2.3.3 was reached for all constitutive laws within 10 iterations. It is worth mentioning, that the MFF using the Klotz curve as a target did not work for the isotropic neo-Hookean and Mooney–Rivlin materials. Though sometimes used for cardiac mechanics [15,73], also Hadjicharalambous et al. [34] reported previously that with these constitutive laws the Klotz curve could not be reproduced. This is suggestive of such materials being inappropriate to model cardiac tissue. The problem for the fitting in our case is that the PV response with these materials results in a concave curve – as also observed in [34, Figure 12] – while the Klotz curve is convex. Hence, the presented MFF failed, however, our approach might still work with these materials for other applications than cardiac mechanics with target functions that are not necessarily convex.

We could further show that the MFF approach can be used with different FE formulations such as P1-P0 elements and locking-free stabilized P1-P1 and MINI elements. Here, the locking-free elements resulted in material parameters that correspond to a stiffer material as the fitting compensates for locking effects in the P1-P0-element formulation. As expected, run-times are significantly higher for locking-free elements; still the efficiency of the MFF approach allowed for unloading and parameter estimation within reasonable time frames.

### 4.4 Goodness of fit and sensitivity analysis

We were able to show that our method allows to match the predicted volume of the unloaded geometry $V_{\text{0}}^{\text{klotz}}$ and the given end-diastolic volume $V_{\text{ed}}^{\text{dat}}$ almost perfectly while reproducing the shape of the PV-curve within uncertainty of the empiric Klotz relation (Table 3). It is important to note that the EDPVR cannot be accurately determined *in vivo* for a number of fundamental limitations. First, the LV volume during diastolic filling depends on the pressure difference between LV cavity and intra-pericardial pressure and is also influenced by the presence of time-varying non-zero active stresses. Time-varying intra-pericardial pressure is non-negligible and influenced by intra-thoracic pressure modulated by breathing and, as such, cannot be recorded easily beyond specifically designed experiments [38,37]. Further, tails of the active stress transients during diastole depend on cytosolic calcium, the state of Troponin-C buffering as well as the spatially varying sarcomere geometries. None of these factors can be monitored. As such, the time course of active stresses during diastole must be considered unknown, albeit efforts have been invested to estimate these [91]. The Klotz relation has been shown to be robust across species and a range of pathological conditions under *ex vivo* conditions where both volume and intra-cavitary pressure can be controlled with higher accuracy, but, owing to its empirical nature, must be considered an approximation of the EDPVR in an individual patient under *in vivo* conditions.

Independently of the uncertainties a Klotz-based surrogate EDPVR is afflicted with, the application to the patient cohort resulted in excellent fittings to the Klotz curve for all cases with a goodness of fit measure *r*^*A_n_*,rel^ ranging between 7.38% and 14.77%. Also *r*^*V*_0_,rel^ was very low for all cases, although a small trend towards better fits in the CoA cases was observed. Note that only for these cases the measured pressure was available, see also Section 3.3.1.

The influence of the input pressure $p_{\text{ed}}^{\text{dat}}$ was noticeable, resulting in different shapes of the Klotz EDPVR (Figs. 8 and 9) and therefore in different material parameters. This influenced the goodness of fit to some extent, hence, the sensitivity to this measure should be considered, especially in the case when invasively measured pressures are not available.

Fiber orientations are known to have a great impact on cardiac mechanics simulations [25,71], thus, the influence of variations of the fiber angle *α* on fitting performance is an integral point of the sensitivity analysis. Variations of fiber angles resulted in very similar outcomes in terms of goodness of fit as well as computational cost. The altered stiffness due to altered fiber directions was compensated by the variation of material parameters, hence resulting in similar PV-curves.

Changes in initial guesses for model parameters were not reflected in the fitting outcome at all and resulted in the same fitted material parameters and curves. Only the run-times for unloading varied when using initial guesses lying further from the final result. This robustness of the approach is essential for the interpretation of material parameters for characterizing patient pathology and understanding changes in material properties under HF conditions.

The fitting outcome was not sensitive to variations of the bulk modulus *κ* when using locking-free elements. However, for P1-P0-elements the shape error increased for higher values of *κ*, compensating for the increased locking of the P1-P0-elements. As in all simulations modeling nearly-incompressible behavior of soft tissue, special care has to be taken when setting this parameter for simple linear elements.

Finally, the choice of the model function (8) was motivated by the mathematical structure of the constitutive laws and the Klotz relation. In Appendix D, we could demonstrate that several other model functions can be used for the fitting, all leading to excellent results. As the MFF approach is applicable to many kinds of target loading curves other than the Klotz relation, the definite choice of the model function is specific to the problem.

### 4.5 Limitations

First, the workflow presented in this paper relies on the empiric Klotz relation. However, the approach also works in the exact same fashion for other experiment-based PV-curves, discrete data points obtained from loading experiments, or experimental/clinical *in vivo* measurements of the EDPVR.

Second, as residual strain is not considered, in general, this method does not generate a uniform fiber-stretch field which is generally assumed at end-diastolic state [14,23,65]. Heterogeneity in fiber stretch in an end-diastolic state impairs the Frank-Starling mechanism, as shown in a recent modeling study [8]. In future studies, the presented workflow will be extended by using ideas based on growth and remodeling [27,88] to address this issue.

Third, we presented the methodology only for single-chamber LV models, while solid- and electromechanical whole-organ simulations of the heart are becoming feasible [9,11,72,78]. Nevertheless, the workflow was successfully tested for bi-ventricular models [42] as well as 4-chamber models. Here, we used one homogeneous material for the ventricles and default values from the literature for the atria, e.g., [7], and applied patient-specific pressures in the different chambers for the forward solve in Algorithms 1 and 2. We did not present these simulations as an empirical estimation for the EDPVR was only available for the LV given as the Klotz law. To the best of our knowledge, no comparable approximation of EDPVR exists for the right ventricle or the atria.

Finally, we cannot provide a rigorous proof that the resulting unloaded configuration and material parameter sets are unique. However, we could show that the method was robust with respect to initial values leading to the same result for all simulations, see Section 4.4. Further, as we are fitting a strictly convex function (8), we can assume that the Levenberg–Marquardt algorithm results in a unique parameter set [13]. Since the MFF method requires that the estimated parameters are close to this solution, see Equation (14), we can expect that the MFF method is robust with regard to material parameters.

## 5 Conclusion

We report on a novel MFF approach combining the fitting passive mechanical parameters of the myocardium to the shape of the EDPVR and the simultaneous estimation of the unloaded reference configuration. The algorithm only requires image or mesh data from one time instance during diastolic filling and a single measured PV data point of the EDPVR as inputs. The MFF is efficient, robust, and versatile and can be applied to reproduce clinically-relevant PV relationships for patient-specific LV anatomical models within clinically easily feasible time frames in a fully automated fashion. Thus, the method constitutes a further step forward towards a realistic representation of LV passive mechanical function. As such, the MFF is a pivotal building block in workflows for building computational digital twin models of human cardiac EM function at scale, to facilitate the generation of virtual cohorts in translational applications.

## Supplementary Material

Appendix

## Figures and Tables

**Table 1:** Default material parameters for the considered single Fung-type exponential materials. Column 'Model' gives the short name for the constitutive law; 'Ref.' the reference for the parameter fitting; 'Property' the material property specified by the parameter set; and in the following a list of the material parameters. Note that only the scaling parameter *a* has a unit (kPa), all other parameters are dimensionless.

Model	Ref.	Property	Parameters						
			*a* [kPa]	*b*_ff_	*b*_ss_	*b*_nn_	*b*_fs_	*b*_fn_	*b*_ns_
Guccione	[31]	trans.-isotr.	0.876	18.48	3.58	3.58	3.58	1.627	1.627
Usyk	[80]	orthotropic	0.88	5.0	6.0	3.0	10.0	2.0	2.0

**Table 2:** Default material parameters for the considered Holzapfel-Ogden (HO) type materials. Column 'Model' gives the short name for the constitutive law; 'Ref.' the reference for the parameter fitting; 'Property' the material property specified by the parameter set; and in the following a list of the material parameters. Note that parameters *a*_(•)_ have a unit (kPa), while parameters *b*_(•)_ are dimensionless. Model "HO dispersion" includes dispersion of collagen fibers based on (7).

Model	Ref.	Property	Parameters													
			*a* [kPa]	*b*	*a*_f_ [kPa]	*b*_f_	*a*_s_ [kPa]	*b*_s_	*a*_n_ [kPa]	*b*_n_	*a*_fn_ [kPa]	*b*_fn_	*a*_sn_ [kPa]	*b*_fn_	*a*_sn_ [kPa]	*b*_sn_
General HO	[30]	orthotropic	0.180	9.762	2.204	21.597	0.098	49.878	0.508	27.719	1.291	5.295	1.345	2.017	0.947	4.514
Reduced HO	[30]	orthotropic	0.809	7.474	1.911	22.063	–	–	0.227	34.802	0.547	5.691	–	–	–	–
Original HO	[39]	orthotropic	0.33	9.242	18.535	15.972	2.564	10.446	–	–	0.417	11.602	–	–	–	–
HO dispersion	[33]	orthotropic	0.4	6.55	3.05	29.05	1.25	36.65	–	–	0.15	6.28	–	–	–	–
One fiber HO	[39]	trans.-isotr.	0.809	7.474	1.911	22.063	–	–	–	–	–	–	–	–	–	–
Demiray	[16]	isotropic	1.0	6.5	–	–	–	–	–	–	–	–	–	–	–	–

**Table 3:** Fitting results for all *N* = 19 cases are listed in terms of final scaling parameters, *a*_scale_ and *b*_scale_ after convergence of the algorithm, and measures of goodness of fit, $r^{V_{0,\text{rel}}}$ and $r^{A_{n,\text{rel}}}$, along with input parameters, $V_{\text{ed}}^{\text{dat}}$ and $p_{\text{ed}}^{\text{dat}},$ used to compute the Klotz EDPVR. Mean values and standard deviation (SD) of goodness of fit measures were computed separately for each etiology. The whole set of fitted parameters for these cases is given in Table C.11.

Case-ID	Input Parameters		Fitted Parameters		Goodness of Fit	
	$V_{\text{ed}}^{\text{dat}}[\text{ml}]$	$p_{\text{ed}}^{\text{dat}}[\text{kpa}]$	*a*_scale_	*b*_scale_	$r^{V_{0,\text{rel}}}[\%V_{\text{ed}}^{\text{dat}}]$	$r^{A_{\text{n},\text{rel}}}[\%A_{\text{klotz}}]$
01-CoA	155.65	1.75	0.3418	0.5988	0.16	10.00
02-CoA	230.23	2.56	0.5776	0.5671	0.18	11.87
03-CoA	96.48	0.72	0.2111	0.5054	0.16	8.34
04-CoA	92.86	0.61	0.1308	0.6887	0.16	9.86
05-CoA	152.83	1.49	0.3278	0.5458	0.17	9.69
06-CoA	159.27	1.07	0.2576	0.6258	0.13	7.38
07-CoA	123.72	1.09	0.3651	0.5387	0.11	8.39
				Mean (SD)	0.15 (0.03)	9.36 (1.47)
01-AS	239.95	2.80	0.3422	0.4496	0.15	14.22
02-AS	275.31	2.80	0.2953	0.5647	0.16	14.29
03-AS	220.46	2.80	0.2972	0.5081	0.20	14.77
04-AS	97.27	2.80	0.1550	0.6813	0.18	14.71
05-AS	175.89	2.80	0.2511	0.6868	0.17	14.41
06-AS	237.83	2.80	0.2947	0.5526	0.17	14.55
07-AS	120.33	2.80	0.2116	0.5774	0.18	14.77
08-AS	281.70	2.80	0.3204	0.7124	0.15	14.10
09-AS	128.09	2.80	0.2427	0.5729	0.16	14.47
10-AS	208.75	2.80	0.3303	0.5043	0.16	14.41
11-AS	154.64	2.80	0.2406	0.6297	0.16	14.46
12-AS	169.14	2.80	0.1840	0.6567	0.17	14.54
				Mean (SD)	0.17 (0.02)	14.48 (0.21)

**Table 4:** Fitting results for case 06-CoA and 03-AS using different material laws are shown in terms of fitted scaling parameters, *a*_scale_ and *b*_scale_, and measures of goodness of fit, *r*^*V*_0_,rel^ and *r*^*A_n_*,rel^.

	Case 06-CoA				Case 03-AS			
	Fitted Parameters		Goodness of Fit		Fitted Parameters		Goodness of Fit	
Material Model	*a*_scale_	*b*_scale_	*r* ^*V*_0_,rel^ $\left[\%V_{\text{ed}}^{\text{dat}}\right]$	*r*^*A_n_*,rel^ [%*A*_klotz_]	*a*_scale_	*b*_scale_	*r* ^*V*_0_,rel^ $\left[\%V_{\text{ed}}^{\text{dat}}\right]$	*r*^*A_n_*,rel^ [%*A*_klotz_]
Guccione	0.0863	1.8189	0.16	7.44	0.1235	1.4884	0.21	14.74
Usyk	0.0607	3.9060	0.19	7.65	0.1068	2.7406	0.24	15.81
General HO	0.2630	0.5892	0.11	7.27	0.3034	0.4702	0.20	14.76
Reduced HO	0.2576	0.6258	0.13	7.38	0.2972	0.5081	0.20	14.77
Original HO	0.0473	0.8441	0.17	7.59	0.0615	0.6707	0.22	14.79
HO dispersion	0.2973	0.6781	0.14	7.65	0.3554	0.5354	0.23	14.83
One fiber HO	0.2649	0.6382	0.12	7.74	0.2934	0.5518	0.19	14.50
Demiray	0.5050	1.0502	0.16	7.49	0.4955	0.9411	0.14	14.52

**Table 5:** Fitting results for case 06-CoA and 03-AS using P1-P0-elements and stabilized P1-P1-element are compared in terms of fitted scaling parameters and measures of goodness of fit.

	Case 06-CoA				Case 03-AS			
	Fitted Parameters		Goodness of Fit		Fitted Parameters		Goodness of Fit	
Elem. Type	*a*_scale_	*b*_scale_	*r* ^*V*_0_,rel^ $\left[\%V_{\text{ed}}^{\text{dat}}\right]$	*r*^*A_n_*,rel^ [%*A*_klotz_]	*a*_scale_	*b*_scale_	*r* ^*V*_0_,rel^ $\left[\%V_{\text{ed}}^{\text{dat}}\right]$	*r*^*A_n_*,rel^ [%*A*_klotz_]
P1-P0	0.2576	0.6258	0.13	7.38	0.2972	0.5081	0.20	14.77
P1-P1 stab	0.3800	0.5386	0.08	8.36	0.4260	0.4512	0.19	13.93

**Table 6:** Fitting results for case 03-AS are compared for varying $p_{\text{ed}}^{\text{dat}}$ in terms of fitted parameters and measures of goodness of fit.

		Fitted Parameters		Goodness of Fit	
$p_{\text{ed}}^{\text{dat}}$	Deviation	*a*_scale_	*b*_scale_	*r* ^*V*_0_,rel^	*r* ^*A_n_*,rel^
[*kPa*]				$\left[\%V_{\text{ed}}^{\text{dat}}\right]$	[%*A*_klotz_]
2.52	−10%	0.3410	0.4985	0.21	12.34
2.80	0%	0.2972	0.5081	0.20	14.77
3.08	+10%	0.4903	0.3803	0.22	7.68

**Table 7:** The influence of change in fiber orientation on fitting results in terms of fitted parameters and goodness of fit is listed.

Fiber Orientation	Fitted Parameters		Goodness of Fit	
	Deviation	a_scale_	b_scale_	r^V_0_,rel^	r^A_n_,rel^
				$\left[\%V_{\text{ed}}^{\text{dat}}\right]$	[%A_klotz_]
*α*_epi_ =−45°, *α*_endo_ = 45°	−25%	0.2768	0.5005	0.19	14.68
*α*_epi_=−60°, *α*_endo_= 60°	0%	0.2972	0.5081	0.20	14.77
*α*_epi_=−75°, *α*_endo_= 75°	+25%	0.3207	0.5161	0.20	14.84

**Table 8:** Fitting results for case 03-AS are compared for different values of *k* using P1-P0-elements and stabilized P1-P1-elements.

	P1-P0-elements				P1-P1-elements			
	Fitted Parameters		Goodness of Fit		Fitted Parameters		Goodness of Fit	
*k*	*a*_scale_	*b*_scale_	*r* ^*V*_0_,rel^	*r* ^*A_n_*,rel^	*a*_scale_	*b*_scale_	*r* ^*V*_0_,rel^	*r* ^*A_n_*,rel^
[*kPa*]			$\left[\%V_{\text{ed}}^{\text{dat}}\right]$	[%*A*_klotz_]			$\left[\%V_{\text{ed}}^{\text{dat}}\right]$	[%*A*_klotz_]
default	0.2972	0.5081	0.20	14.77	0.4260	0.4512	0.19	13.93
1000	0.2614	0.5199	0.22	15.26	0.4266	0.4511	0.19	13.93
3000	0.1149	0.6263	0.30	18.08	0.4264	0.4511	0.19	13.92
5000	0.0340	0.8113	0.41	20.15	0.4266	0.4511	0.19	13.94

**Table 9:** Fitting results in terms of fitted scaling parameters and measures of goodness of fit for the two example cases executed using the reduced HO constitutive law with parameters from literature [39] (no init) as initial guess and with an MFF initialization step (init). Outcomes of the CFF method, optimizing one, two, or three parameters, *n*_opt_ = {1, 2, 3}, are listed with number of iterations (it.) of the Nelder–Mead algorithm.

		Case 06-CoA						Case 03-AS					
			Fitted Parameters			Goodness of Fit			Fitted Parameters			Goodness of Fit	
Mode	*n*_opt_	it.	*a*_scale_	$\left[b_{\text{scale}}^{\text{iso}}\right]$	$\left[b_{\text{scale}}^{\text{aniso}}\right]$	*r* ^*V*_0_,rel^	*r* ^*A_n_*,rel^	it.	*a*_scale_	$\left[b_{\text{scale}}^{\text{iso}}\right]$	$\left[b_{\text{scale}}^{\text{aniso}}\right]$	*r* ^*V*_0_,rel^	*r* ^*A_n_*,rel^
						$\left[\%V_{\text{ed}}^{\text{dat}}\right]$	[% *A*_klotz_]					$\left[\%V_{\text{ed}}^{\text{dat}}\right]$	[% *A*_klotz_]
init	1	17	0.3475	0.5000	0.5000	0.027	11.13	9	0.2982	0.5000	0.5000	0.024	14.72
	2	46	0.2365	0.6493	0.6493	0.002	8.30	73	0.2588	0.5252	0.5252	0.001	15. 43
	3	94	0.2371	0.6827	0.6274	0.001	8.20	78	0.2571	0.5524	0.5144	0.002	15.59
no init	1	20	0.3477	0.5000	0.5000	0.025	11.15	22	0.2969	0.5000	0.5000	0.016	14.72
	2	41	0.5427	0.3255	0.3255	0.009	28.13	80	0.2587	0.5252	0.5252	0.000	15. 40
	3	130	0.2382	0.7321	0.5948	0.008	8.43	167	0.2588	0.5979	0.4881	0.001	15.03
