# Supplementary material for: Robust and efficient fixed-point algorithm for the inverse elastostatic problem to identify myocardial passive material parameters and the unloaded reference configuration
Source: J Comput Phys. Author manuscript; Available in PMC 2022 Aug 1. (PMC7612790; doi:10.1016/j.jcp.2022.111266)
Supplement: Appendix [file EMS145159-supplement-Appendix.pdf]

Appendix A. Established algorithms to find the unloaded reference configuration

Unloading algorithms as proposed by Sellier [75] in Algorithm 3 and by Rausch et al. [68] in Algorithm 4.

Algorithm 3 Sellier's Inverse Method [75].

initialize $\boldsymbol{X}^0 = \boldsymbol{x}^{\text{dat}}$; $k = 0$
do
  solve forward problem, $\boldsymbol{x}^k = \boldsymbol{\phi}\left(\boldsymbol{X}^k\right)$
  calculate nodal error vector, $\boldsymbol{R}^k = \boldsymbol{x}^k - \boldsymbol{x}^{\text{dat}}$
  update reference vector, $\boldsymbol{X}^{k+1} = \boldsymbol{X}^k - \boldsymbol{R}^k$
  compute maximal nodal error, $r^{\|\boldsymbol{x}\|_\infty} = \max_{i \in [1, N_{\text{nodes}}]} \left\| \boldsymbol{x}_i^k - \boldsymbol{x}_i^{\text{dat}} \right\|_2$
  update counter, $k = k + 1$
while $r^{\|\boldsymbol{x}\|_\infty} \geq \epsilon$
unloaded reference configuration, $\boldsymbol{X}^* = \boldsymbol{X}^k$

Algorithm 4 Augmented Sellier's Inverse Method [68].

initialize $\boldsymbol{X}^0 = \boldsymbol{x}^{\text{dat}}$; $k = 0$; $\beta = 1.0$
do
  solve forward problem, $\boldsymbol{x}^k = \boldsymbol{\phi}\left(\boldsymbol{X}^k\right)$
  calculate nodal error vector, $\boldsymbol{R}^k = \boldsymbol{x}^k - \boldsymbol{x}^{\text{dat}}$
  if $k > 0$ **then**
    update augmentation parameter, $\beta = -\beta \dfrac{\boldsymbol{R}^{k-1} : \left[\boldsymbol{R}^k - \boldsymbol{R}^{k-1}\right]}{\left[\boldsymbol{R}^k - \boldsymbol{R}^{k-1}\right] : \left[\boldsymbol{R}^k - \boldsymbol{R}^{k-1}\right]}$
  end if
  update reference vector, $\boldsymbol{X}^{k+1} = \boldsymbol{X}^k - \beta \boldsymbol{R}^k$
  compute maximal nodal error, $r^{\|\boldsymbol{x}\|_\infty} = \max_{i \in [1, N_{\text{nodes}}]} \left\| \boldsymbol{x}_i^k - \boldsymbol{x}_i^{\text{dat}} \right\|_2$
  update counter, $k = k + 1$
while $r^{\|\boldsymbol{x}\|_\infty} \geq \epsilon$
unloaded reference configuration, $\boldsymbol{X}^* = \boldsymbol{X}^k$

Table A.10
Comparison of unloading algorithms: Iteration numbers and computational times in (HH:MM:SS) format for all 19 cases. Error "–" indicates that the unloading algorithm didn't converge within 50 iterations.

	Algorithm 3, [75]		Algorithm 4, [68]		Algorithm 1	
	$r^{\|\boldsymbol{x}\|_\infty} < 1.0$	$r^{\|\boldsymbol{x}\|_\infty} < 0.1$	$r^{\|\boldsymbol{x}\|_\infty} < 1.0$	$r^{\|\boldsymbol{x}\|_\infty} < 0.1$	$r^{\|\boldsymbol{x}\|_\infty} < 1.0$	$r^{\|\boldsymbol{x}\|_\infty} < 0.1$
01-CoA	6 (0:13:39)	10 (0:23:24	5 (0:11:07)	6 (0:13:40)	5 (0:12:36)	6 (0:13:07)
02-CoA	7 (0:16:17)	16 (0:37:31	5 (0:10:38)	9 (0:20:21)	6 (0:13:39)	10 (0:21:59)
03-CoA	–	–	6 (0:05:11)	9 (0:06:32)	5 (0:03:34)	7 (0:05:03)
04-CoA	5 (0:07:16)	9 (0:15:10)	5 (0:07:12)	6 (0:09:10)	4 (0:05:59)	6 (0:08:55)
05-CoA	5 (0:08:52)	11 (0:20:39)	5 (0:08:17)	6 (0:09:41)	4 (0:06:26)	6 (0:09:51)
06-CoA	5 (0:12:34)	8 (0:19:51)	5 (0:13:18)	6 (0:14:21)	3 (0:07:36)	6 (0:15:23)
07-CoA	5 (0:04:55)	9 (0:08:59)	5 (0:04:50)	9 (0:09:22)	5 (0:05:02)	9 (0:09:00)
01-AS	–	–	6 (0:12:42)	10 (0:21:00)	6 (0:12:06)	10 (0:21:17)
02-AS	–	–	6 (0:21:44)	9 (0:30:02)	5 (0:18:08)	9 (0:30:17)
03-AS	50 (2:25:48)	–	8 (0:32:51)	–	5 (0:16:22)	14 (0:49:07)
04-AS	–	–	7 (0:11:23)	10 (0:21:15)	6 (0:12:18)	8 (0:17:33)
05-AS	15 (0:48:38)	29 (1:37:08)	5 (0:16:22)	7 (0:24:52)	5 (0:17:02)	7 (0:24:23)
06-AS	–	–	8 (0:27:13)	–	6 (0:21:12)	18 (1:03:50)
07-AS	–	–	7 (0:11:54)	10 (0:20:02)	6 (0:12:36)	8 (0:14:45)
08-AS	–	–	6 (0:30:05)	–	5 (0:23:31)	14 (1:08:48)
09-AS	23 (0:38:21)	35 (0:45:59)	6 (0:09:10)	9 (0:14:15)	6 (0:09:36)	8 (0:12:49)
10-AS	–	–	6 (0:13:54)	–	6 (0:14:20)	10 (0:23:49)
11-AS	8 (0:16:48)	17 (0:32:28)	5 (0:10:40)	7 (0:14:42)	5 (0:11:01)	7 (0:15:30)
12-AS	–	–	6 (0:18:21)	9 (0:24:47)	5 (0:16:09)	7 (0:23:31)

Appendix B. Klotz relation

Considering the limited availability of clinical data of the EDPVR, the computational method proposed by Klotz et al. [45] is utilized, which enables prediction of the EDPVR by a single measured PV-pair. According to this seminal work the volume of the unloaded geometry V_0^{klotz} of the LV can be empirically determined by

$$V_0^{\text{klotz}} = V_{\text{ed}}^{\text{dat}} \left(0.6 - 0.006 p_{\text{ed}}^{\text{dat}}\right), \tag{B.1}$$

where $V_{\text{ed}}^{\text{dat}}$ and $p_{\text{ed}}^{\text{dat}}$ is a measured PV-pair at end-diastole. Further, the EDPVR is described by the power law

$$p = \alpha V^{\beta}, \tag{B.2}$$

where p is the cavity pressure in mmHg, V is the cavity volume in mL, and the constants α and β are defined by the relations

$$\alpha = \frac{30}{\left(V_{30}^{\text{klotz}}\right)^{\beta}} \quad \text{and} \quad \beta = \frac{\log\left(p_{\text{ed}}^{\text{dat}}/30\right)}{\log\left(V_{\text{ed}}^{\text{dat}}/V_{30}^{\text{klotz}}\right)}. \tag{B.3}$$

Here, V_{30}^{klotz} is the estimated cavity volume at a pressure of 30 mmHg, given by

$$V_{30}^{\text{klotz}} = V_0^{\text{klotz}} + \frac{V_{\text{ed}}^{\text{dat}} - V_0^{\text{klotz}}}{\left(p_{\text{ed}}^{\text{dat}}/A_{\text{n}}\right)^{1/B_{\text{n}}}}, \tag{B.4}$$

where A_{n} and B_{n} were determined empirically as 27.78 mmHg and 2.76 respectively. Equation (B.3) requires that $p_{\text{ed}}^{\text{dat}} \leq$ 22 mmHg (2.93 kPa), which applies to all patient cases in the CARDIOPROOF cohort (see Table 3).

Appendix C. Full parameter list for the reduced Holzapfel–Ogden law

Table C.11
Fitted parameters for all $N = 19$ cases for the reduced Holzapfel–Ogden law; for this default material parameters given in Table 2 are multiplied according to Equation (10) with final scaling parameters given in Table 3.

Case-ID	a [kPa]	b	a_{f} [kPa]	b_{f}	a_{n} [kPa]	b_{n}	a_{fs} [kPa]	b_{fs}
default	0.8090	7.4740	1.9110	22.0630	0.2270	34.8020	0.5470	5.6910
01-CoA	0.2765	4.4754	0.6532	13.2113	0.0776	20.8394	0.1870	3.4078
02-CoA	0.4673	4.2385	1.1038	12.5119	0.1311	19.7362	0.3159	3.2274
03-CoA	0.1708	3.7774	0.4034	11.1506	0.0479	17.5889	0.1155	2.8762
04-CoA	0.1058	5.1473	0.2500	15.1948	0.0297	23.9681	0.0715	3.9194
05-CoA	0.2652	4.0793	0.6264	12.0420	0.0744	18.9949	0.1793	3.1061
06-CoA	0.2084	4.6772	0.4923	13.8070	0.0585	21.7791	0.1409	3.5614
07-CoA	0.2954	4.0262	0.6977	11.8853	0.0829	18.7478	0.1997	3.0657
01-AS	0.2768	3.3603	0.6539	9.9195	0.0777	15.6470	0.1872	2.5587
02-AS	0.2389	4.2206	0.5643	12.4590	0.0670	19.6527	0.1615	3.2137
03-AS	0.2404	3.7975	0.5679	11.2102	0.0675	17.6829	0.1626	2.8916
04-AS	0.1254	5.0920	0.2962	15.0315	0.0352	23.7106	0.0848	3.8773
05-AS	0.2031	5.1331	0.4799	15.1529	0.0570	23.9020	0.1374	3.9086
06-AS	0.2384	4.1301	0.5632	12.1920	0.0669	19.2316	0.1612	3.1448
07-AS	0.1712	4.3155	0.4044	12.7392	0.0480	20.0947	0.1157	3.2860
08-AS	0.2592	5.3245	0.6123	15.7177	0.0727	24.7929	0.1753	4.0543
09-AS	0.1963	4.2819	0.4638	12.6399	0.0551	19.9381	0.1328	3.2604
10-AS	0.2672	3.7691	0.6312	11.1264	0.0750	17.5506	0.1807	2.8700
11-AS	0.1946	4.7064	0.4598	13.8931	0.0546	21.9148	0.1316	3.5836
12-AS	0.1489	4.9082	0.3516	14.4888	0.0418	22.8545	0.1006	3.7373

Appendix D. Choice and comparison of model functions

As default we chose the model function in Equation (8) for its similarity to the constitutive laws and to keep $b \neq 0$. Nevertheless, the fitting method also worked for other model functions, e.g., those listed below:

$$\Phi_1(x, x_0) = \frac{a}{2b}\left\{\exp\left[b\left(\frac{x - x_0}{x_0}\right)\right] - 1\right\}, \tag{energy-Demiray}$$

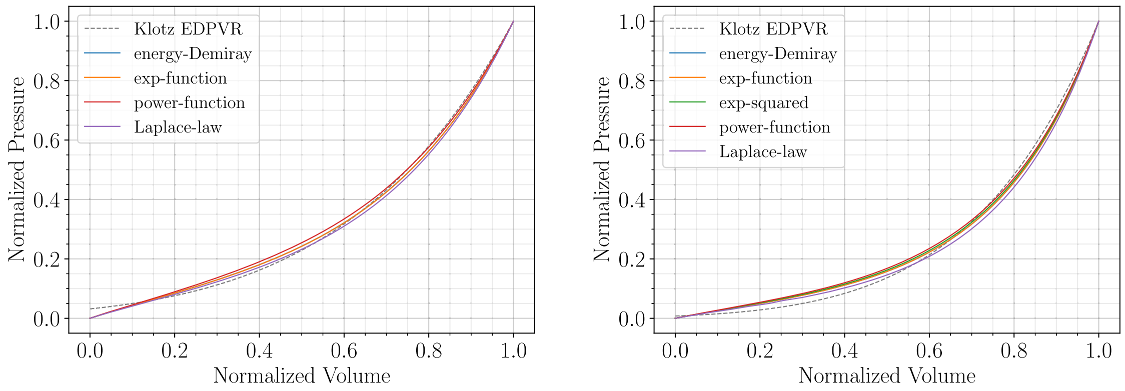

Fig. D.13. Fitting results of case 06-CoA (left) and 03-AS (right) using different model functions are shown. The normalized Klotz EDPVR (dashed gray) is visualized along with the respective fitted curves.

$$\Phi_2(x,x_0) = a\left\{\exp\left[b\left(\frac{x-x_0}{x_0}\right)\right]-1\right\}, \qquad \text{(exp-function)}$$

$$\Phi_3(x,x_0) = \frac{a}{2b}\left\{\exp\left[b\left(\frac{x-x_0}{x_0}\right)^2\right]-1\right\}, \qquad \text{(exp-squared)}$$

$$\Phi_4(x,x_0) = a\left(\frac{x-x_0}{x_0}\right)^b, \qquad \text{(power-function)}$$

$$\Phi_5(x,x_0) = a\left(\frac{x-x_0}{x_0}\right)\exp\left[b\left(\frac{x-x_0}{x_0}\right)^2\right]\left[\left(\frac{x+v_{\text{wall}}}{x}\right)^{2/3}-1\right], \qquad \text{(Laplace-law)}$$

with parameters a and b. Similar to Section 2.3.2, a Levenberg–Marquardt least-squares algorithm was used (i) to fit the model function $\Phi_i(x,x_0)$ to the Klotz relation, with x the volumes as predicted by the Klotz power law (B.2) and x_0 the volume of the unloaded geometry volume V_0^{klotz} (B.1); and (ii) to fit the model function $\Phi_i(x,x_0)$ to the re-loading curve in the current step k of the unloading algorithm, with x the volumes at the different loading points and x_0 the cavitary volume of the current reference configuration $\boldsymbol{X}^k$. All model functions are designed such that $\Phi(x_0,x_0)=0$ to ensure loading curves that have zero pressure at the unloaded reference configuration with volume V_0. The first model function (energy-Demiray), the same as in Equation (8), was inspired by the constitutive law of Demiray (3); the second (exp-function) is a standard exponential fitting function; the third (exp-squared) was inspired by the anisotropic contributions in the HO models (6); the fourth (power-function) is related to the Klotz power law (B.2); and the fifth (Laplace-law) was inspired by an extension of the Laplace law [53] to take the volume of the LV wall, v_{wall}, into account:

$$\boldsymbol{\sigma} = \frac{p}{\left(\frac{x+v_{\text{wall}}}{x}\right)^{2/3}-1},$$

where the stress tensor $\boldsymbol{\sigma}$ at pressure p is computed from the constitutive law (exp-squared) above

$$\boldsymbol{\sigma} = \frac{\partial\Phi_3(x,x_0)}{\partial\lambda}, \quad \lambda := \left(\frac{x-x_0}{x_0}\right).$$

Here, x is a substitute for the volume of the cavity at pressure p for the Klotz law and the reloading, respectively; x_0 a substitute for the reference volume; and λ a strain-like value.

Results The unloading and parameter estimation was performed as in Section 3.1 for cases 06-CoA and 03-AS. Normalized fitting results for all different model functions and both cases are shown in Fig. D.13 and Table D.12. We see that all model functions work well for case 03-AS with only minor differences in the goodness of fit and the fitted parameters. For case 06-CoA the fitting with the (exp-squared) model did not converge and the fitting with the power-function showed a considerably slower convergence compared to the other model functions. Differences between (energy-Demiray) and (exp-function) are very small, both show fast convergence and excellent fitting results, rendering these model functions a favorable choice for the fitting. We noticed for all cases, also visible in Fig. D.13, that the fitting with the (Laplace-law) model function gives results that are closest to the Klotz curve in the lower pressure range but further afar in the higher pressure range, overall resulting in the largest values of the area error $r^{A_n,\text{rel}}$. However, the (Laplace-law) fitting always had the lowest deflection error defined as

Table D.12
Fitting results for case 06-CoA and 03-AS using different model functions are shown in terms of fitted scaling parameters and measures of goodness of fit.

Model Function	Case 06-CoA				Case 03-AS			
	Fitted Parameters		Goodness of Fit		Fitted Parameters		Goodness of Fit	
	a_{scale}	b_{scale}	$r^{V_0,\text{rel}}$ [$\%V_{\text{ed}}^{\text{dat}}$]	$r^{A_n,\text{rel}}$ [$\%A_{\text{klotz}}$]	a_{scale}	b_{scale}	$r^{V_0,\text{rel}}$ [$\%V_{\text{ed}}^{\text{dat}}$]	$r^{A_n,\text{rel}}$ [$\%A_{\text{klotz}}$]
energy-Demiray	0.2576	0.6257	0.13	7.39	0.2971	0.5076	0.19	14.77
exp-function	0.2558	0.6267	0.11	7.36	0.2968	0.5078	0.19	14.77
exp-squared	-	-	-	-	0.3120	0.4956	0.16	14.64
power-function	0.2878	0.6076	0.59	7.14	0.3260	0.4721	0.19	14.59
Laplace-law	0.2358	0.6587	0.28	8.40	0.2605	0.5391	0.25	15.55

$$r^{\text{shape}} = \max_{p\in[0,p_{\text{ed}}]} \left| V^{\text{klotz}}(p) - V^{\text{sim}}(p) \right|. \tag{D.1}$$

Hence, for certain cases it can be a good alternative to the (energy-Demiray) function.

Model functions Ψ_i given above are only a small subset of functions that we tried for our fitting to the Klotz curve. All of them showed satisfying results and convergence rates but other functions might work as well for the procedure. Since the Klotz curve resembles an exponential function, it is not surprising that the (energy-Demiray) and (exp-function) worked best. However, as our method would work for all kinds of target EDPVR other than the Klotz law, the choice of the model function is specific to the problem.
